# Source apportionment and source-specific risk evaluation of potential toxic elements in oasis agricultural soils of Tarim River Basin

**DOI:** 10.1038/s41598-023-29911-3

**Published:** 2023-02-20

**Authors:** Yizhen Li, Jilili Abuduwaili, Long Ma, Wen Liu, Tao Zeng

**Affiliations:** 1 State Key Laboratory of Desert and Oasis Ecology, Xinjiang Institute of Ecology and Geography, Chinese Academy of Sciences, Urumqi, 830011 China; 2 Research Center for Ecology and Environment of Central Asia, Chinese Academy of Sciences, Urumqi, 830011 China; 3 University of Chinese Academy of Sciences, Beijing, 100049 China

**Keywords:** Environmental chemistry, Geochemistry, Ecology

## Abstract

As rapidly developing area of intensive agriculture during the past half century, the oases in the source region of the Tarim River have encountered serious environmental challenges. Therefore, a comparative analysis of soil pollution characteristics and source-specific risks in different oases is an important measure to prevent and control soil pollution and provide guidance for extensive resource management in this area. In this study, the concentration of potential toxic elements (PTEs) was analyzed by collecting soil samples from the four oases in the source region of the Tarim River. The cumulative frequency curve method, pollution index method, positive matrix factorization (PMF) model, geographical detector method and health risk assessment model were used to analyze the pollution status and source-specific risk of potential toxic elements in different oases. The results showed that Cd was the most prominent PTE in the oasis agricultural soil in the source region of the Tarim River. Especially in Hotan Oasis, where 81.25% of the soil samples were moderately contaminated and 18.75% were highly contaminated with Cd. The PTEs in the Hotan Oasis corresponded to a moderate level of risk to the ecological environment, and the noncarcinogenic risk of soil PTEs in the four oases to local children exceeded the threshold (TH > 1), while the carcinogenic risk to local residents was acceptable (1E−06 < TCR < 1E−04). The research results suggested that the Hotan Oasis should be the key area for soil pollution control in the source region of the Tarim River, and agricultural activities and natural sources, industrial sources, and atmospheric dust fall are the priority sources that should be controlled in the Aksu Oasis, Kashgar Oasis and Yarkant River Oasis, respectively. The results of this study provide important decision-making support for the protection and management of regional agricultural soil and the environment.

## Introduction

Potential toxic element (PTE) pollution in the soil is a major threat to global soil health^1^. Agricultural soil is especially closely related to human health. With the rapid development of intensive agricultural activities, PTE pollution in agricultural soil has attracted worldwide attention because of its serious harm to the quality and safety of agricultural products, the health of agricultural ecosystems and human health^2–4^.

The enrichment factor (*EF*), ground accumulation index (*I*_*geo*_) and pollution load index (*PLI*) are commonly used to quantify the impact of human activities on the concentration of pollutants such as PTEs in agricultural soil^5–7^. The main reason for using these pollution indices is to determine the natural baseline of pollutants. In general, the background values (BVs) and geochemical baseline values (GBVs) are used as the natural baselines of pollutants^8^. Due to natural variability and extensive human input, it is practically impossible to accurately quantify true BVs^9^. In contrast, the GBV composition comprises geologically natural concentrations (natural background) and diffuse anthropogenic contributions, which can better reflect the natural change in element concentrations in topsoil^10,11^. Therefore, since the concept of GBVs was proposed (IGCP 3601993), an increasing number of studies have used this measure as the natural baseline for assessing PTE pollution^9,10,12–14^. However, although the GBVs of PTEs have been established in many areas in China, there has been no report on the GBVs of PTEs in oases with intensive human activities in the source region of the Tarim River.

There are many different sources of PTEs in soil, which complicates the prevention and treatment of soil pollution. Meanwhile, previous studies have shown that PTEs from different sources differ in their bioavailability and geochemical components, which can lead to different risk levels^15^. Therefore, to save time, labor and material resources, it is of great relevance for soil pollution prevention and management to determine the priority controlled sources by assessing the risk levels of PTEs from different sources^16^. Therefore, Taghvaee et al. proposed a source-specific risk assessment method based on source analysis and risk assessment methods to carry out this work^17^. In recent years, source-specific risk assessment methods have attracted increasing attention, and have been widely applied in the analysis of various pollutants in air particulate matter^18^, heavy metals in road dust^16^, potential toxic elements in agricultural soil^19^ and pollutants in river sediments^20^. The key to source-specific risk assessment is the source apportionment of PTEs. At present, methods for analyzing the source of PTEs in soil mainly include chemical mass balance (CMB), the UNMIX model, absolute principal component score-multiple linear regression (APCS-MLR)^21–23^ and positive matrix factorization (PMF)^24^, etc. However, most of the above models and methods are mathematical in terms of the appearance and intrinsic properties. The interpretation of the source of pollutants is based on the judgment of the evaluator, and the results of source analysis are highly subjective^25^. In recent years, an increasing number of studies have combined geographic detector models to analyze the sources of pollutants in the environment, because it can capture the spatial relationship between various driving factors and pollutant concentrations^26–28^. With geographical detection results, the results of recognition by receptor models such as the PMF model can be interpreted and verified objectively, and comprehensive and accurate conclusions can be obtained.

The Tarim River Basin is one of the major water-scarce areas in the world. This basin contains fragile ecosystems, and it is also the largest inland basin in China. Against the background of the western development strategy, this region has been devoted to commodity cotton production^29^. The intensive agriculture in the oasis system has brought major environmental challenges, such as land desertification and soil salinization, to the Tarim River Basin, resulting in substantial reliance on the application of nitrogen, phosphorus and potassium fertilizers to improve crop yield^30,31^. In addition, the Taklimakan Desert in the Tarim River Basin is an important source of dust in this region and beyond. When dust passes through areas with dense human activities, it combines with local pollutants and brings pollutants to the surface through wet and dry deposition. Therefore, the source region of the Tarim River has been affected by intense human activities and adverse climatic conditions, and the pressure for sustainable environmental development in this region is high^32^, therefore this area of dense human activities in an arid inland river basin of Northwest China is an ideal area to assess the PTE pollution of soil. However, until now, investigations and research on the PTEs of agricultural soil in the source region of the Tarim River Basin in Xinjiang have typically been carried out at the county^33–36^, small watershed and subregion spatial scales^37–39^, while a systematic analysis of the distribution characteristics of PTEs and pollution characteristics of oasis agricultural soil in different regions at large spatial scales is lacking. Thus, the comprehensive assessment and countermeasures of oasis farmland pollution have been hindered. Therefore, it is of great value and necessity to comprehensively evaluate the source specific risk of agricultural soil PTEs in the source region of the Tarim River. With that in mind, the purpose of this study is to (1) analyze the pollution level of soil PTEs in different oasis farmlands according to GBVs, (2) allocate the potential sources of PTEs by comparing the principal component analysis (PCA) method, PMF model and geographical detector method, and (3) combine the source apportionment with ecological and human health risk assessment to quantify the source-specific risks of soil PTEs in different oases. Because no previous study has discussed the GBVs and source-specific risks of PTEs in the soil of the source region of the Tarim River, the results of this study will be helpful for regional monitoring of the impact of PTE pollution in agricultural soil and for formulating appropriate control and management strategies for agricultural soil environmental pollution.

## Materials and methods

### Study area

**Figure 1:**
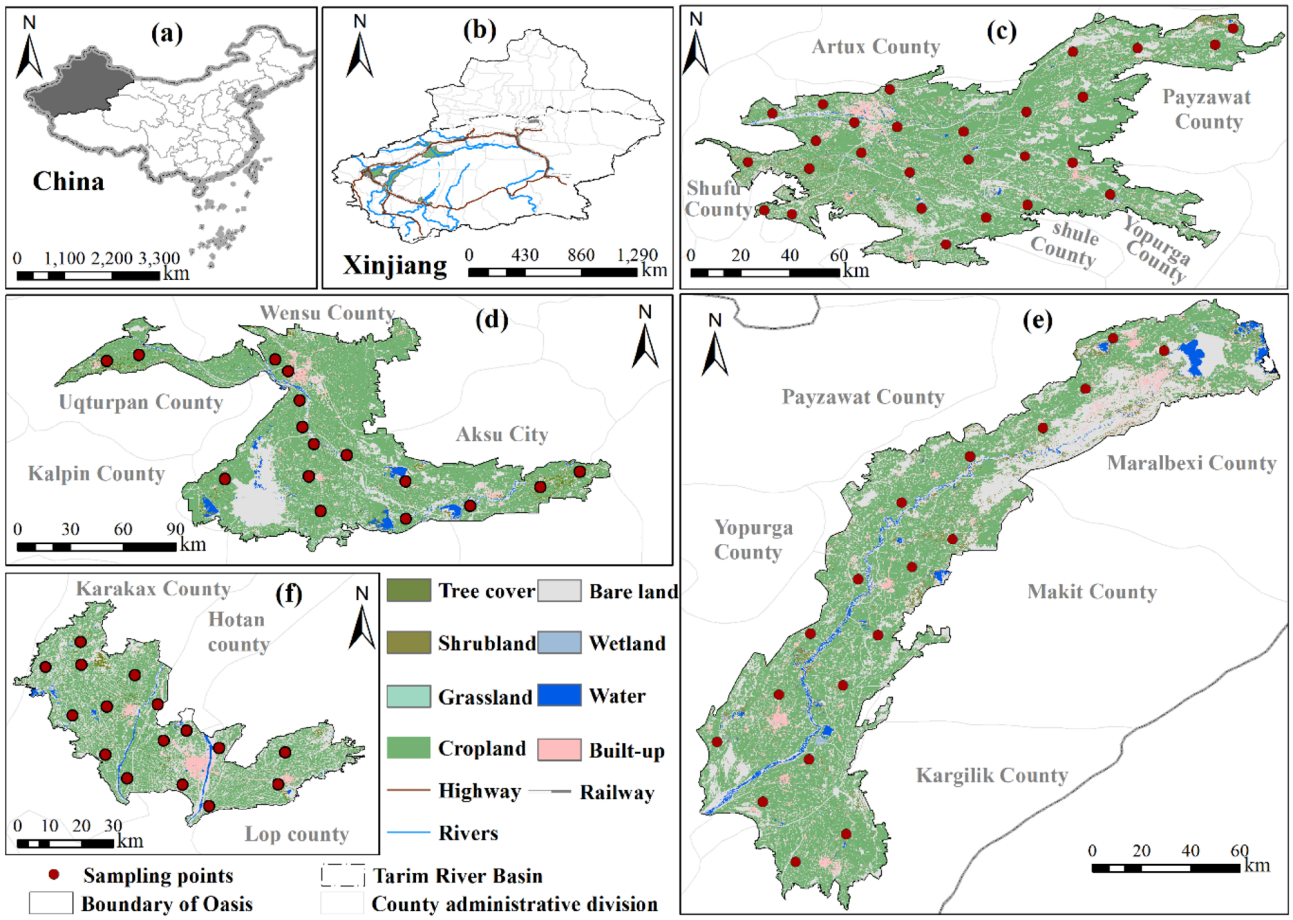
Sampling locations in the Kashgar Oasis (**c**), Aksu Oasis (**d**), Yarkant River Oasis (**e**) and Hotan Oasis (**f**). The graphs were generated by QGIS 3.26.3 (https://www.qgis.org) and the review number of China Map is GS (2020) 4619, the Land use data are from the ESA global 30 m resolution land use cover dataset (https://viewer.esa-worldcover.org/worldcover). The combination of graphs (**a**–**e)** was accomplished with linkscape 1.2.1 (https://inkscape.org).

The Tarim River is located in the center of Eurasia and crosses the Taklimakan Desert, the second largest desert in the world. It is the longest inland river in China and the main river in the Xinjiang Uygur Autonomous Region in Northwest China^40^. Moreover, as the most important water supply in southern Xinjiang, the main stream and tributaries of the Tarim River connect scattered oases around the Taklimakan Desert, providing water to more than 8 million people in southern Xinjiang^41^. The runoff of the Tarim River mainly comes from its three tributaries, namely the Aksu River, Yarkant River and Hotan River. The source region of the Tarim River is the area covered by these three tributaries^42^. In this study, the Yarkant River Oasis, Kashgar Oasis, Aksu Oasis and Hotan Oasis in the source region of the Tarim River were selected as the study areas (Fig. 1c–f).

The economy of the oases in the source region of the Tarim River is dominated by animal husbandry and agriculture, mainly grain and cotton, and the region is an important base for high-quality cotton and production in Xinjiang. Since the 1950s, the Tarim River Basin has become one of the most important resettlement-receiving areas and farming bases in China. Large-scale population growth and agricultural production have placed great pressure on the water and land resources in the region^42^. Since the 1990s, due to the impact of high-intensity agricultural development, the total area of arable land in the oasis in the source region of the Tarim River has continued to increase, and the areas of the Aksu Oasis and Yarkant River Oasis have expanded considerably^43^. With the increases in population size and cultivated land area, the application intensity and density of nitrogen and phosphorus fertilizers in the Tarim River Basin are also increasing. At present, the amount of fertilizer applied in the basin accounts for approximately 60% of the total for all of Xinjiang, and the amount is increasing at an annual rate of more than 5%. Meanwhile, the fertilizer density (393.60 kg/hm^2^) has been higher than the average level of all of Xinjiang, and has far exceeded the globally recognized safety limit (225 kg/hm^2^)^44^.

### Sample collection

In this study, soil sampling was carried out in four oases (Yarkant River Oasis, Kashgar Oasis, Aksu Oasis and Hotan Oasis) in the source region of the Tarim River in June 2021. A hand-held GPS instrument was used for field sampling, and samples were sealed in polyethylene zip-lock bags and marked with sample number and location. The specific distribution information of the sampling points is shown in Supplementary Table S1 and Fig. 1. According to the soil survey specifications, when collecting soil samples, an equal mixture of soil from 3 to 5 subsamples was collected from each sample site, for a total of approximately 1.0–1.5 kg samples. A total of 78 topsoil samples (sampling depth 0–20 cm) and 78 subsoil samples (sampling depth 60–80 cm) were collected.

### Sample processing and PTE determination

The soil samples returned to the laboratory were naturally aired and air-dried to remove debris such as rocks, roots, and animal and plant residues, and then each dry powder sample was sieved through a 200-mesh sieve. Finally, an appropriate amount of sample was sent to Aussie Analytical Testing (Guangzhou) Co., Ltd., for PTE determination.

When the PTEs were determined, the sample was digested with perchloric acid, nitric acid, hydrofluoric acid and hydrochloric acid, and then diluted hydrochloric acid was used to determine the volume. Finally, elemental analysis was carried out by inductively coupled plasma emission spectrometry (ICP-AES, model Agilent 5110, origin USA) and inductively coupled plasma-mass spectrometry (ICP-MS, model Agilent 7900, origin USA). After the spectral interference between elements was corrected, the analysis results of PTE (As, Cd, Co, Cu, Ni, Pb, Sb, Sn, Tl, V and Zn) contents were obtained.

For the determination of PTEs, measures such as inserting monitoring substances (inserting blank samples, duplicate samples and reference materials in each test batch), using the same or different methods for repeated testing, repeatedly testing the retained samples, analyzing the relevant characteristics of different results of samples and blind sample testing were adopted to carry out quality monitoring and ensure the accuracy of the results. Accuracy and precision were controlled according to the following requirements: relative deviation (*RD*) < 10% and relative error (*RE*) < 10%. The detection limits of As, Cd, Co, Cu, Ni, Pb, Sb, Sn, Tl, V and Zn were 0.2, 0.02, 0.1, 0.2, 0.2, 0.5, 0.05, 0.2, 0.02, 1 and 2 µg/g, respectively.

### Analysis methods

#### Determination of GBVs and calculation of pollution level

Elemental GBVs refer to the concentration level of elements in the absence of environmental pollution. The most widely used method to determine the GBVs of soil elements is the cumulative frequency curve method^8,10,19^. Since subsoil is considered to be less affected than other soil levels by human activities, PTE concentrations in subsoil (60–80 cm) are usually used to estimate GBVs. The specific principle and steps of determining the elemental GBVs by using the cumulative frequency distribution curve method are as follows^8^: (1) Before drawing the element cumulative frequency distribution curve, the Kolmogorov–Smirnov (K–S) test is used to detect whether the element concentration data conform to a normal distribution. If the element concentration data do not conform to a normal distribution, the data need to be transformed. (2) The cumulative frequency distribution curve is drawn with the cumulative frequency and element concentration as the X and Y axes, respectively, and extreme values are gradually eliminated to meet the criteria of *p* < 0.05 and *R*^2^ > 0.95 in linear regression^45^. (3) The inflection point of the cumulative frequency distribution curve is observed. If there is an inflection point in the curve, the mean value of the concentration data below the inflection point is defined as the GBV. If there are two inflection points in the curve, the mean value of the concentration data below the lower inflection point is defined as the GBV. If there is no inflection point in the curve, the mean value of all the bottom soil element concentration data is the GBV.

To explore the pollution level of PTEs, the pollution factor (*PF*), pollution load index (*PLI*) and Nemerow comprehensive pollution index (*P*_*N*_) of oasis agricultural soil PTEs were calculated based on the calculated GBVs as follows^19,46,47^:

1 $$PF = \frac{{C_{i} }}{{B_{i} }},$$

2 $$PLI = \sqrt[n]{{PF_{1} \times PF_{2} \times \cdots \times PF_{n} }},$$

3 $$P_{N} = \sqrt {\frac{{PLI_{average}^{2} + PLI_{\max }^{2} }}{2}} ,$$

where, *C*_*i*_ is the PTE concentration investigated in the soil samples, *B*_*i*_ is the GBV of the corresponding PTE, and *n* is the number of PTEs. The *PF*, *PLI* and *P*_*N*_ associated with specific pollution types are presented in Supplementary Table S2.

### Source-specific risk assessment

#### PMF model

As one of the source analysis models recommended by the U.S. Environmental Protection Agency (EPA), the PMF model has been widely used for source analysis of pollutants such as heavy metals and PTEs^24,48,49^. The PMF model first uses the weight to determine the error in the chemical components of the acceptor, and then iteratively determines the pollution source and its contribution ratio by the least square method, which has advantages in the source analysis of pollutants in the environment^50^. In this study, USEPA PMF5.0 was used to quantitatively analyze the source resolution of soil PTEs. The calculation process and principles of PMF 5.0 are described in Supplementary Material S1. In addition, SPSS 20 was used to conduct correlation analysis and PCA of associations between PTEs and qualitatively identify their potential sources. The factor detectors in geographic detectors were also used to help determine the specific sources of PTEs in agricultural soil. The principle and calculation method are presented in Supplementary Material S1.

### Distribution of PTE concentrations from different sources

After obtaining the calculation results of the PMF model, the concentration of a PTE in the soil of a specific source at each point was calculated as follows^46,51^:

4 $$C_{ij}^{k} = C_{ij}^{k*} \times C_{i} ,$$

where *C*^*k*^_*ij*_ is the concentration of the *j*th PTE from the *k*th source in the *i*th sample, µg/g; *C*^k*^_ij_ is the calculated contribution rate of the *j*th PTE from the *k*th source in the *i*th sample, and *C*_*i*_ is the concentration of soil PTE in the *i*th sample, µg/g.

### Source-specific ecological risk assessment

According to the concentration of soil PTE from specific sources, combined with the ecological risk assessment method recommended by Men et al.^16^, the specific-source ecological risk of oasis agricultural soil PTEs in the source region of the Tarim River was calculated as follows:

5 $$ER_{ij}^{k} = \frac{{C_{ij}^{k} }}{{B_{i} }} \times T,$$

6 $$EIRI_{ij}^{k} = \sqrt {\frac{{ER_{ij\max }^{k\,2} + ER_{ij\,average}^{k\,2} }}{2}} ,$$

where, *ER*^*k*^_*ij*_ is the ecological risk of the *j*th PTE from the *k*th source at the *i*th sampling point and T is the toxicity coefficient of the PTE (the toxicity coefficients of As, Cd, Co, Cu, Ni, Pb, Sb, Sn, Tl, V and Zn are 10, 30, 5, 5, 5, 10, 1, 10, 2 and 1, respectively)^52,53^. *EIRI*^*k*^_*ij*_ is the ecological risk of multiple PTEs from the *k*th source at the *i*th sampling point, and the risk classification standard is shown in Supplementary Table S2.

### Source-specific human health risk assessment

Combining the health risk assessment model developed by the US Environmental Protection Agency^54^ with concentrations of PTEs from different sources, the health risks of a specific-source of PTEs to children (under 6 years of age) and adults (over 18 years of age) can be calculated. In view of the fact that the study subjects were soil samples, this study considered only human health risks due to hand-to-mouth ingestion and dermal contact. According to the Manual of Exposure Factors^55,56^, the average daily dose (ADD) of these two exposure routes was calculated^51^:

7 $$ADD_{{ij_{ing} }}^{k} = \frac{{C_{ij}^{k} \times R_{ing} \times EF \times ED}}{BW \times AT} \times 10^{ - 6} ,$$

8 $$ADD_{{ij_{der} }}^{k} = \frac{{C_{ij}^{k} \times SA \times AF \times ABS \times EF \times ED}}{BW \times AT} \times 10^{ - 6} ,$$

where, *ADD*_*ij*_^*k*^_*ing*_ is the *ADD* of the *j*th PTE from the *k*th source at the *i*th sampling point through the hand-to-mouth ingestion route, mg/kg·d, and *ADD*_*ij*_^*k*^_*der*_ is the *ADD* of the *j*th PTE from the *k*th source at the *i*th sampling point through the dermal contact route, mg/kg·d, The definitions and specific reference values of other parameters in the model are shown in Supplementary Table S3.

According to the obtained *ADD* of a PTE, the total hazard index (*THI*) and total carcinogenic risk (*TCR*) were used to quantify the total noncarcinogenic risk and total carcinogenic risk, respectively. The specific calculation method was as follows^54,57^:

9 $$HI = \frac{{ADD_{{ij_{ing} }}^{k} }}{{R_{f} D_{ing} }} + \frac{{ADD_{{ij_{der} }}^{k} }}{{R_{f} D_{der} }},$$

10 $$THI = \sum {HI} ,$$

11 $$CR_{ij}^{k} = ADD_{{ij_{ing} }}^{k} \times SF_{ing} + ADD_{{ij_{der} }}^{k} \times SF_{der} ,$$

12 $$TCR = \sum {CR_{ij}^{k} } .$$

The specific definitions and values of *R*_*f*_*D*_*ing*_, *R*_*f*_*D*_*der*_, *SF*_*ing*_ and *SF*_*der*_ of each element in the equation are shown in Supplementary Table S4. If the THI value is less than 1, there is no significant risk of noncarcinogenic effects; if the THI value is greater than 1, there is noncarcinogenic risk. TCR is the probability of developing any cancer due to exposure to carcinogenic hazards, and its acceptable level is 1 × 10^–6^ to 1 × 10^–4^^58^.

### Statistical analysis

The maximum, minimum, mean, standard deviation (SD), coefficient of variation (Cv) and other descriptive statistical indicators of PTEs were calculated by SPSS 25. The K-S test was used to determine whether the PTE concentration data fit a normal distribution.

## Results

### Concentrations of PTEs in agricultural soil of oases

The descriptive statistical results of 11 PTE concentrations in agricultural soils of the four oases are shown in Table 1. The concentration order of different PTEs in the four oases was different. In terms of mean values, the concentrations of Co, Cu, Ni, Pb, Sb, V and Zn in Kashgar Oasis agricultural soil were higher than those in the other three oases, and the concentrations of Sn and Tl in Hotan Oasis agricultural soil were the highest. The Cv value of As in the Hotan Oasis was the highest (0.31), indicating that compared with that in other oases and other PTEs, the As in Hotan Oasis soil was more likely to have external inputs other than natural sources^59^. According to the "Soil Environmental Quality · Agricultural Land Soil Pollution Risk Control Standard (Trial)" (GB15618-2018)^60^, the corresponding elements of oasis agricultural soil in the source region of the Tarim River did not exceed the pollution risk control standard. Compared with the soil environmental quality provisions in the "Environmental Quality Assessment Standard for Producing Areas of Edible Agricultural Products" (HJ332-2006)^61^, the corresponding elements in the soil of the four oases were also below the limits.

**Table 1:** Descriptive statistics of PTEs of oasis agricultural soil in the source region of the Tarim River.

PTEs		As	Cd	Co	Cu	Ni	Pb	Sb	Sn	Tl	V	Zn
Yarkant River Oasis	Min	9.40	0.12	7.50	13.80	18.30	17.10	0.82	2.20	0.47	53.00	53.00
	Max	18.20	0.19	11.20	24.50	29.40	22.20	1.26	3.10	0.63	73.00	84.00
	Mean	13.28	0.16	9.13	17.74	22.18	18.83	1.00	2.59	0.54	61.42	66.05
	SD	2.40	0.02	1.02	3.08	3.37	1.35	0.11	0.21	0.04	5.46	8.68
	Cv	0.18	0.12	0.11	0.17	0.15	0.07	0.11	0.08	0.07	0.09	0.13
Kashgar Oasis	Min	9.30	0.10	7.90	13.50	17.70	14.90	0.94	1.70	0.43	52.00	51.00
	Max	16.20	0.22	15.10	33.80	39.80	24.10	1.58	3.50	0.83	94.00	110.0
	Mean	12.79	0.17	11.03	22.68	26.59	19.94	1.16	2.46	0.57	68.96	76.74
	SD	1.82	0.03	1.56	3.80	4.84	2.34	0.17	0.53	0.11	8.95	12.58
	Cv	0.14	0.20	0.14	0.17	0.18	0.12	0.15	0.22	0.19	0.13	0.16
Aksu Oasis	Min	7.80	0.14	8.20	15.20	19.90	14.80	0.87	1.90	0.40	54.00	56.00
	Max	19.00	0.26	12.60	27.10	30.90	20.60	1.94	2.80	0.55	76.00	86.00
	Mean	13.13	0.18	9.95	21.24	24.44	17.13	1.34	2.39	0.49	62.19	72.19
	SD	2.73	0.04	1.09	3.21	2.96	1.44	0.28	0.29	0.05	5.97	9.35
	Cv	0.21	0.19	0.11	0.15	0.12	0.08	0.21	0.12	0.09	0.10	0.13
Hotan Oasis	Min	7.70	0.11	7.60	15.00	18.90	15.30	0.83	2.10	0.46	53.00	49.00
	Max	20.00	0.18	10.30	21.80	29.50	18.60	1.06	3.90	0.70	66.00	81.00
	Mean	11.69	0.14	9.25	18.55	24.48	16.96	0.92	2.85	0.58	60.94	64.88
	SD	3.66	0.02	0.82	2.05	3.05	1.11	0.07	0.68	0.08	3.77	7.82
	Cv	0.31	0.13	0.09	0.11	0.12	0.07	0.07	0.24	0.14	0.06	0.12
RSV (GB15618-2018) (pH > 7.5)		25	0.6	–	100	190	170	–	–	–	–	300
HJ332-2006 (pH > 7.5)		20	0.4		100	60	50					300
Bortala River Watershed^3^		20.03	0.24	9.72	24.07	24.99	17.93	–	–	–	72.87	77.36
Yili River Watershed^3^		12.03	0.23	10.43	22.72	24.00	19.10	–	–	–	77.53	70.38
Jiuquan, Hexi Corridor^62^		–	–	–	37.2	39.9	–	–	–	–	83.2	57.3
Wuwei, Hexi Corridor^63^		12.66	–	–	28.76	41.14	21.80	–	–	–	62.92	60.81
Bahariya Oasis, Egypt^64^					70.91		8.35				808.78	123.53

Compared with the PTE concentrations in the agricultural soil of oases in other arid areas, the concentrations of Cd, Cu and V in the soil of the study area were lower than those of the Bortala River Basin and Ili River Basin^3^ in the northern Tianshan Mountains, while the concentrations of Co, Ni, Pb and Zn were similar to those of corresponding elements in agricultural soil of the Bortala River Basin and Ili River Basin^3^. Compared with the concentrations of PTEs in agricultural soil of Wuwei and Jiuquan cities in the Hexi Corridor^62,63^, the concentrations of Cu, Ni, Pb and V elements in agricultural soil of the four oases in the study area were lower. Similarly, the concentrations of Cu, V and Zn in the study area were much lower than the corresponding concentrations in the agricultural soil of the Bahariya Oasis, Egypt^64^.

### Geochemical baseline values of PTEs in oasis agricultural soils

The PTE concentration of the subsoil (60–80 cm) after logarithmic and reciprocal conversion passed the KS normal distribution test (*p* > 0.05). The GBVs of PTEs in agricultural soils of the four oases in the source region of the Tarim River were calculated according to the cumulative frequency method, and the results are shown in Supplementary Figs. S1–4 and Table 2. The GBVs of PTEs in agricultural soils were very different among the four oases. For example, except for those of Ni, Sn, Tl and V, the GBVs of PTEs in agricultural soils of the Hotan Oasis were much lower than those of the other three oases (Table 2). There was also a large gap between the GBVs of PTEs in agricultural soil and the soil background values in China^65^, Xinjiang^66,67^ and Xinjiang agricultural land^66^. In particular, the GBV of Zn obtained in this study was 3.6–5 times the soil background value of Xinjiang agricultural land. Under the same land use type, there are differences in the abundance of environmental PTEs due to heterogeneity in the disturbance degree of human activities and the parent material and soil formation processes^68^. Therefore, there were some differences in the GBVs of each PTE among the four oases in this study when the land use types were all agricultural soils.

**Table 2:** GBVs of PTEs in oasis agricultural soils of the source region of the Tarim River.

	As	Cd	Co	Cu	Ni	Pb	Sb	Sn	Tl	V	Zn
Yarkant River Oasis	14.17	0.14	9.26	21.53	19.88	16.92	0.97	2.55	0.48	56.81	84.75
Kashgar Oasis	11.51	0.14	10.40	22.70	25.07	19.46	1.25	2.57	0.57	70.70	73.30
Aksu Oasis	13.19	0.14	8.67	20.93	24.68	17.54	1.58	2.23	0.47	55.29	69.00
Hotan Oasis	7.16	0.05	8.53	16.56	22.94	16.56	0.82	2.23	0.50	62.25	61.00
Background value of China^65^	11.2	0.097	12.7	22.6	26.9	26.0	1.21	2.6	0.62	82.4	74.2
Background values of Xinjiang Province^66,67^	11.2	0.12	15.9	26.7	26.6	19.4	1.08	2.2	0.524	74.9	68.8
Background value of cropland in Xinjiang^66^	9.09	0.12		35.8	26.4	13.5					16.8

### Pollution characteristics of PTEs in agricultural soil of oases

**Figure 2:**
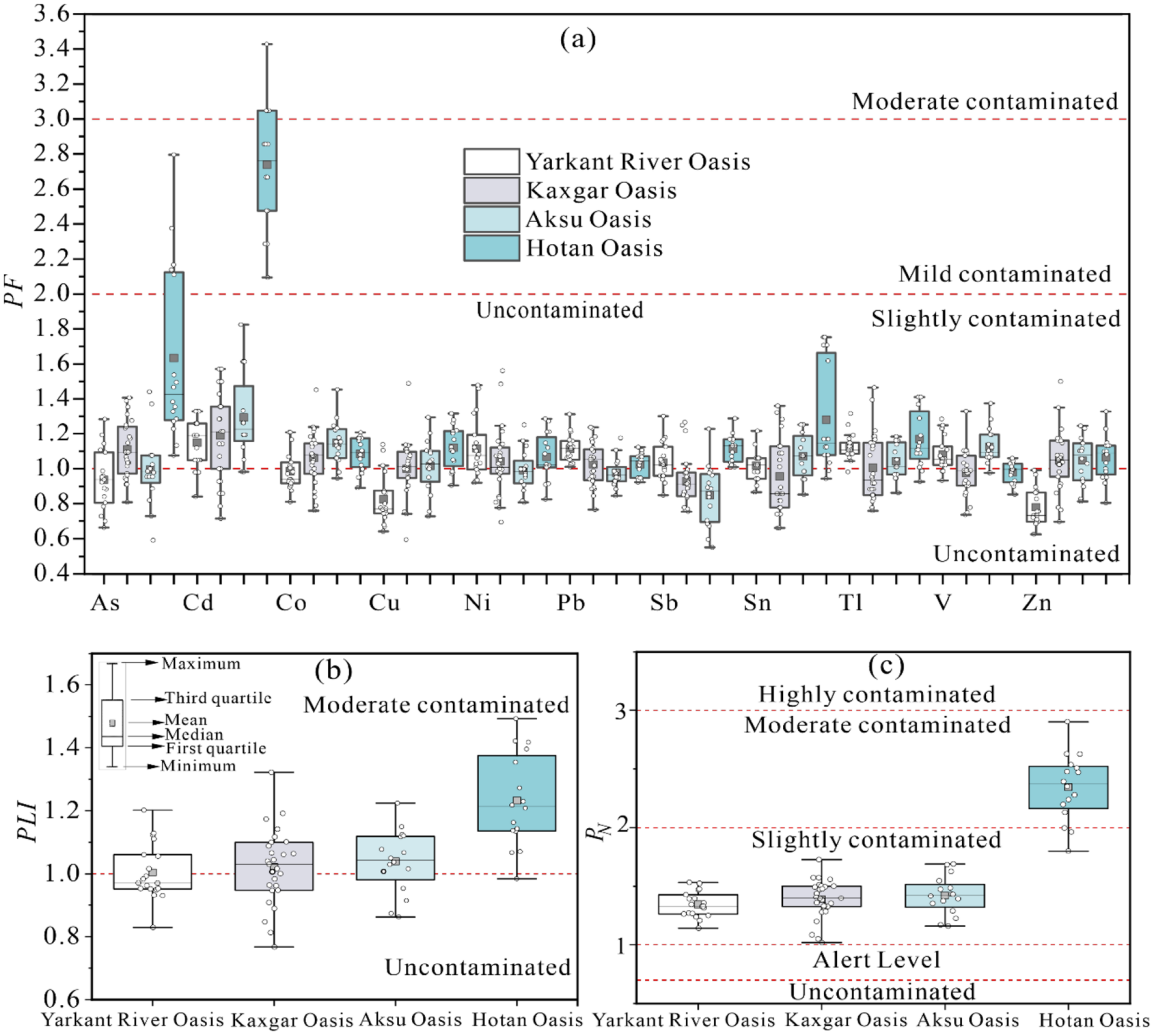
Box-whisker plots of *PF* (**a**), *PLI* (**b**) and *P*_*N*_ (**c**) of PTEs in oasis agricultural soils.

According to the GBVs of the above elements, the PF, PLI and P_N_ of PTEs in agricultural soil of the four oases were calculated, and the results are shown in Fig. 2. The results of *PF* showed that (Fig. 2a), except for As and Cd in the Hotan Oasis, the PTEs of agricultural soils in the four oases were at uncontaminated and slightly contaminated levels, indicating that the oasis agricultural soils in the source region of the Tarim River were affected by human activities to a certain extent. From the perspective of individual elements, the *PF* of Cd in agricultural soils of the four oases was higher than that of other PTEs. Among them, Cd in most soil samples from the Hotan Oasis (81.25%) showed mild contamination, while Cd in the remaining samples (18.75%) showed moderate contamination. Since Cd in soil is generally regarded as an indicator of agricultural activities involving fertilizer use, the results suggest that soil in oases (especially the Hotan Oasis) in the source region of the Tarim River may be seriously affected by agricultural activities.

The results of the pollution load index of multiple PTEs showed that the agricultural soils in the Yarkant River Oasis, Kashgar Oasis and Aksu Oasis were between uncontaminated and moderate contamination levels, and most soil samples in the Hotan Oasis were moderately contaminated. *P*_*N*_ showed similar results. The reason for this result was the high *PF* values of As and Cd in the agricultural soil of the Hotan Oasis (Fig. 2a), which caused the high comprehensive pollution index in this area. It is worth noting that compared with those in the other three areas, most PTEs (As, Cd, Pb, Sb, V and Zn) in the agricultural soil of the Hotan Oasis had lower mean values, but their *PLI* and *P*_*N*_ values were higher. This was closely related to the lower GBVs of PTEs in the Hotan area.

### Source apportionment and source specific risk assessment of PTEs in oasis agricultural soil

#### Source apportionment of soil PTEs

Factor analysis and correlation analysis were used to identify the main sources of PTEs in the main oasis agricultural soil in the source region of the Tarim River. The results are shown in Table 3 and Supplementary Fig. S5. Table 3 shows that PTEs in the agricultural soil of the Yarkant River Oasis had two principal factors. The first principal component F1, which explained 61.6% of the total variance, mainly described Co, V, Cu and Sb and moderately described Cd, Ni and Tl. The second principal component, F2, accounted for 21.8% of the variance and mainly described As, Pb and Sn. There were three main factors for PTEs in Kashgar Oasis agricultural soil. Factor 1, which explained 58.9% of the total variance, was mainly composed of As, Co, Cu, Ni, Pb, Sb, V and Zn; factor 2, which accounted for 24.8% of the variance, was mainly composed of Sn and Tl; and factor 3, which accounted for 7.9% of the variance, was composed of Cd. There were three main factors for the PTEs of Aksu Oasis agricultural soil. Factor 1 was mainly composed of Cd, Co, Cu, Ni, Pb, V and Zn; factor 2 was mainly composed of Sn and Tl; and factor 3 was composed of As and Sb. PTEs in agricultural soil of the Hotan Oasis had only one main factor, which was composed of all (11) elements. To verify the above results of PTE extraction from oasis agricultural soil by PCA, Spearman correlation analysis was performed on PTEs from the soils of the four oases. The results showed that the correlations between PTEs in agricultural soils of the four oases were consistent with the results of PCA, indicating the reliability of the PCA results.

**Table 3:** Principal component analysis results of PTEs of oasis agricultural soil in the source region of the Tarim River.

PTEs	Yarkant River Oasis		Kashgar Oasis			Aksu Oasis			Hotan Oasis
	F1 (61.6%)	F2 (21.8%)	F1 (58.9%)	F2 (24.8%)	F3 (7.9%)	F1 (52.7%)	F2 (22.3%)	F3 (11.5%)	F1 (85.2%)
As	0.441	0.746	0.830	0.347	0.177	0.527	0.447	0.515	0.825
Cd	0.764	0.091	0.347	− 0.714	0.573	0.592	− 0.354	− 0.219	0.761
Co	0.949	− 0.269	0.938	− 0.234	− 0.198	0.938	− 0.017	− 0.310	0.961
Cu	0.915	− 0.346	0.929	− 0.313	− 0.070	0.920	− 0.330	− 0.002	0.989
Ni	0.587	− 0.632	0.665	− 0.533	− 0.444	0.871	− 0.241	− 0.341	0.967
Pb	0.725	0.606	0.823	0.335	0.386	0.728	0.504	− 0.244	0.925
Sb	0.929	0.054	0.873	0.265	0.031	0.702	− 0.115	0.647	0.963
Sn	0.459	0.790	0.526	0.786	− 0.152	0.245	0.923	0.144	0.892
Tl	0.785	0.285	0.585	0.797	0.021	0.438	0.843	− 0.238	0.960
V	0.939	− 0.305	0.889	− 0.300	− 0.252	0.937	− 0.146	− 0.076	0.922
Zn	0.902	− 0.270	0.800	− 0.369	0.184	0.729	− 0.331	0.427	0.963

**Figure 3:**
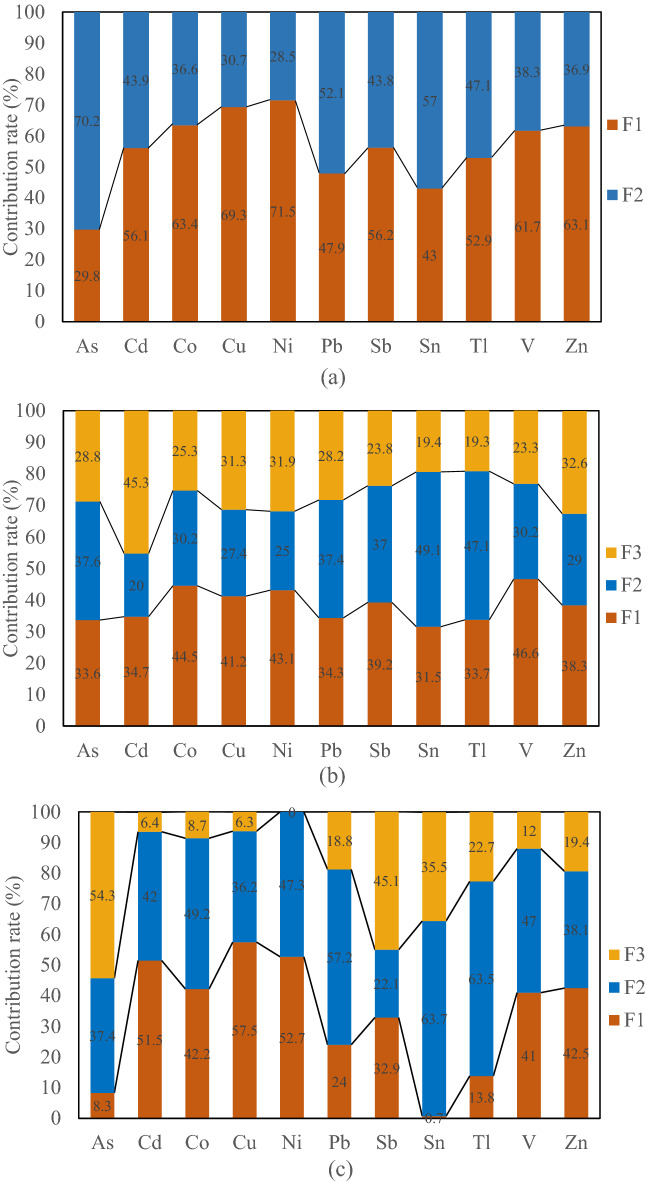
PMF analysis results of PTEs of agricultural soils in the Yarkant River Oasis (**a**), Kashgar Oasis (**b**) and Aksu Oasis (**c**).

To further determine the specific sources and contribution rates of PTEs in oasis agricultural soil in the source region of the Tarim River, the PMF model was adopted for analysis, and the results are shown in Fig. 3. Overall, the classification results of PTEs in agricultural soils of the four oases by the PMF model were consistent with the results of PCA. According to the above results, eight environmental factors related to the source of soil PTEs were selected: distance from factory (*DF*), distance from road (*DR*), *pH*, soil type (*ST*), total nitrogen (*TN*), soil fine silty particle size percentage (*Fine silty*), soil silty particle size percentage (*Silty*), and soil coarse silty particle size percentage (*Coarse silty*). A Geodetector model of PTEs and environmental factors in soil was constructed, and the results are shown in Supplementary Table S5. Among them, the *TN* content in soil was considered to be an index related to the intensity of agricultural activities^69^. Since the particle composition of atmospheric dust in the study area is mainly silt^70^, the silty particle size content of soil was selected as the atmospheric dust index in this study.

In the Yarkant River Oasis, Co, Cu and Zn showed no pollution at most of the sampling points for the first source factor of PTEs in agricultural soil (Fig. 2), indicating that the levels of these elements in soil were less affected by human activities. Meanwhile, the results of the factor detector showed that *ST* was one of the factors explaining the spatial distribution of Co, Cu, Sb, V and Zn (Supplementary Table S5). The Geodetector results revealed *TN* as one of the strong drivers of Cd and Tl, the former of which is found in pesticides and fertilizers and the latter of which may also enter agricultural soil through sewage irrigation and fertilizers contaminated by industrial wastewater^71,72^. Therefore, it is inferred that F1 in the agricultural soil of the Yarkant River Oasis had a mixed source of natural resources and agricultural activities. The three elements in F2 (As, Pb and Sn) were closely related to fuel combustion and traffic factors, among which Pb is usually selected as an identifying element for traffic sources (including leaded exhaust gas, vehicle tires and brake pads)^73^. In the results of the Geodetector model, the best explanatory factors for Pb and Sn were *Silt* and *DR*, while the best explanatory factor for As was *Sli*t. Therefore, F2 was inferred to be the source of road dust/atmospheric dust.

The weights of F1 in Kashgar Oasis agricultural soil PTEs were mainly included As, Co, Cu, Ni, Pb, Sb, V and Zn; the weights of F2 were mainly included Sn and Tl; and F3 were mainly included Cd (Fig. 3b). Similar to the results observed for the Yarkant River Oasis, the factor detector results showed that F1 might have a mixed anthropogenic source, including transportation, industrial and other sources. Both Sn and Tl in F2 are widely used in industrial manufacturing, and Sn can be used as an additive to enhance the properties of steel or alloys^74^. Tl is used in many different industrial manufacturing and medical fields, and metal smelting, sulfuric acid production, coal burning, cement manufacturing and other industrial activities involving the use of Tl minerals are the main pathways by which this element enters the environment^71^. At the same time, the strongest explanatory factor for Sn and Tl in the results of the geographic detector was DF, so F2 was inferred to be an industrial source. F3 contained only Cd (related to agricultural activities), so F3 was inferred to be the source of agricultural activities (Supplementary Table S5).

In the PMF results, the weights of F1 for the PTEs of Aksu Oasis agricultural soil were mainly included Cu, Ni, Cd, Zn, Co, V and Pb; the weights of F2 were mainly included Sn and Tl; and the weights of F3 were mainly included As and Sb (Fig. 3c). Similarly, from the factor detector calculation results, it was inferred that F1 in the Aksu Oasis was a mixed source. According to the results of Geodetector analysis, the best explanatory factors for Sn and Tl in the agricultural soil of the Aksu Oasis were TN and ST. Previous studies have also shown that Sn in soil may also come from agricultural practices (pesticides)^75^, so F2 was inferred to represent agricultural activities and natural sources (rock mineralization). Similarly, according to Supplementary Table S5, F3 was inferred to be the source of agricultural activities. Previous studies have also concluded that agricultural activities such as the application of phosphate fertilizer are also the main source of As and Sb in soil^76,77^, which was consistent with the results of the Geodetector model.

The explanatory factors for the PTEs of agricultural soil in the Hotan Oasis were the total nitrogen content and the silty particle size percentage, indicating that the PTEs in agricultural soil in the Hotan Oasis mainly came from agricultural activities and atmospheric dust fall. The economy in the Hotan Oasis is dominated by irrigated agriculture, and since most oases border large deserts, wind and sand disasters are extremely serious (annual average dust days exceeding 220 days)^78^. The sources of PTEs inferred by the Geodetector model in this study were consistent with the reality.

#### Source-specific ecological risk assessment

**Figure 4:**
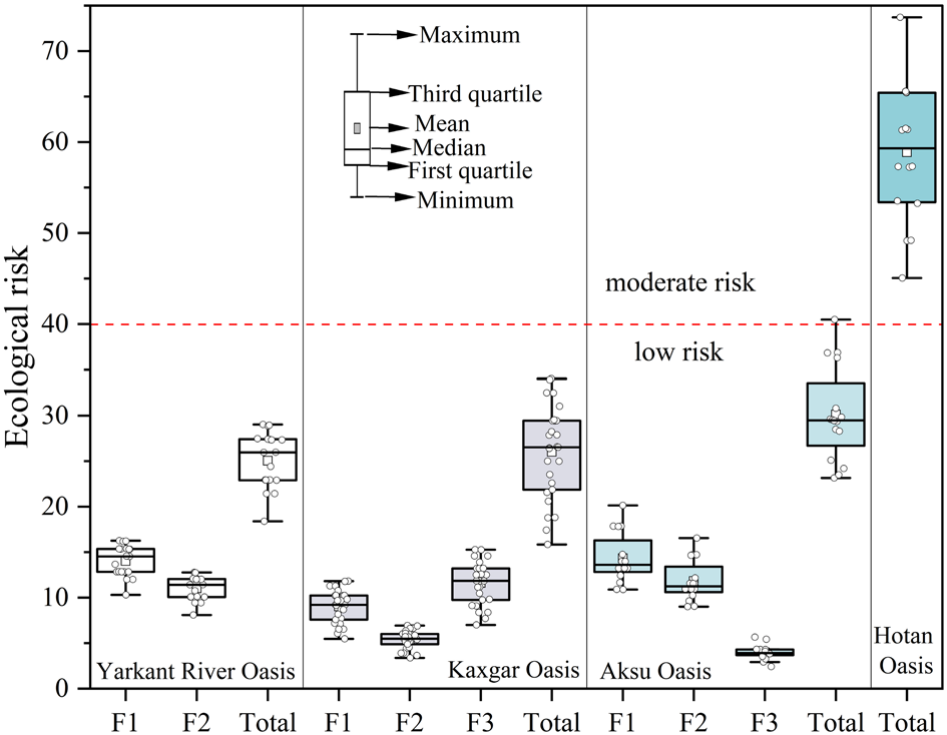
Source-specific ecological risk boxplots of PTEs of agricultural soils in oases in the source region of the Tarim River.

Based on the results of source analysis, the source-specific ecological risks posed by agricultural soil PTEs were evaluated in this study, and the results are shown in Fig. 4. The total ecological risks caused by PTEs in the agricultural soils in the Yarkant River Oasis, Kashgar Oasis and Aksu Oasis were null, while those at of all sampling sites in the Hotan Oasis were moderate risk. According to the results of the source-specific ecological risk of PTEs, there was no direct relationship between the contribution degree of source-specific risks to the total ecological risk and its contribution to the existence of PTEs in soil. In particular, the agricultural activity source (F3), which accounted for only 7.9% of the PTEs in Kashgar Oasis agricultural soil, contributed the most to the total risk, the mixed source (F1), which contributed the most to the existence of PTEs in soil, contributed the second most to the total risk, and the industrial source (F2) contributed the least. The reason for this result was that the toxicity factor of Cd (30) introduced by agricultural activities was significantly higher than that of PTEs released from other sources, which was consistent with the conclusion of other studies^16,19^ that the main source of highly toxic elements was more likely to cause ecological risks than that of low-toxicity elements.

**Figure 5:**
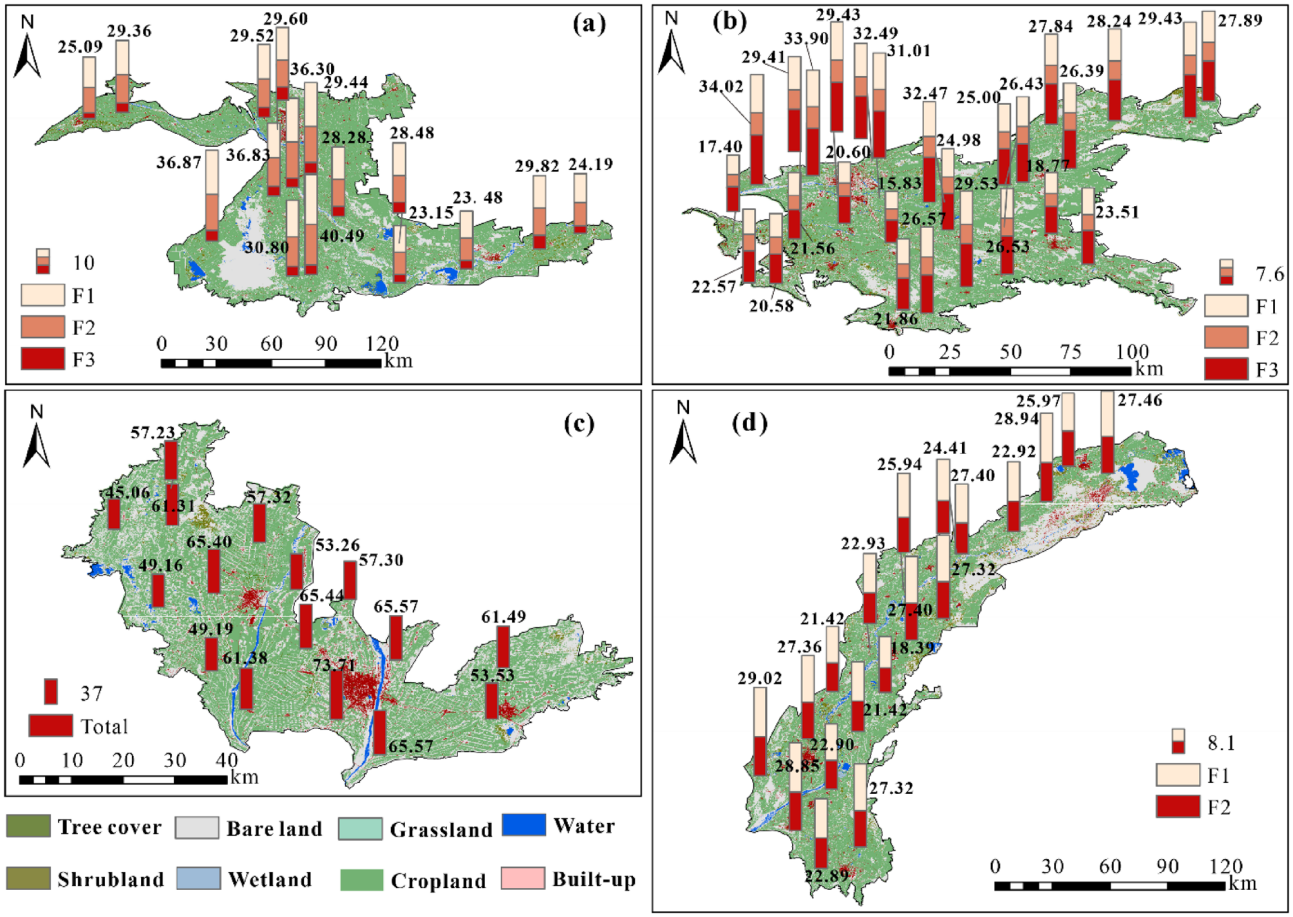
Spatial distribution of the ecological risks of PTEs from different sources in the four oases. The graphs were generated by QGIS 3.26.3 (https://www.qgis.org) and the land use data are from the ESA global 30 m resolution land use cover dataset (https://viewer.esa-worldcover.org/worldcover). The combination of graphs (**a**–**d**) was accomplished with linkscape 1.2.1 (https://inkscape.org).

To explore the spatial distribution of ecological risks generated by PTEs from different sources, a distribution map of ecological risks posed by specific sources at each sampling point was generated (Fig. 5). The total ecological risk of the sampling sites near Awat County in the Aksu Oasis was higher than 40, indicating moderate risk. In addition, the total ecological risk at the other sampling sites was at a low level. However, the ecological risks caused by mixed sources (F1) and agricultural activities and natural sources (F2) were high in the southwestern Aksu Oasis and the sampling sites near Aksu city, resulting in high total ecological risks. The ecological risks at all the sampling sites in the two oases in Kashgar (Kashgar Oasis and Yarkant River Oasis) were low. Notably, the sampling sites near Kashgar city had high total ecological risks due to the high ecological risks caused by PTEs from mixed sources (F1) and agricultural activity sources (F3). The total ecological risk at all sampling sites in the Hotan Oasis was moderate. In particular, the sampling sites near Hotan city had the highest total ecological risk generated by PTEs from agricultural activities and atmospheric dust sources (73.71).

#### Source-specific human health risk assessment

**Figure 6:**
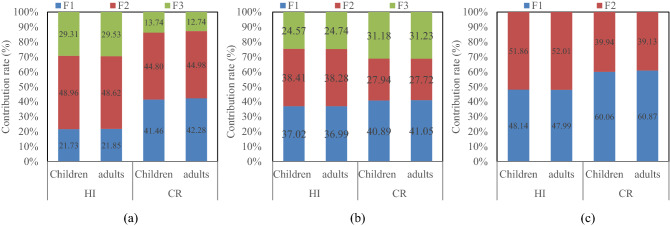
Human health risk proportions of PTEs from different sources in the Aksu Oasis (**a**), Kashgar Oasis (**b**) and Yarkant River Oasis (**c**).

According to the proportions of different sources of each PTE obtained from the PMF model, the source-specific health risks of PTEs in agricultural soil in the four oases to the human body were calculated, and the results are shown in Tables 4 and 5 and Fig. 6. Although the results of noncarcinogenic risk caused by different sources of PTEs in agricultural soil in each oasis showed that adults and children were not at risk, the total THI values of PTEs for children in the four regions were all greater than 1, indicating that soil PTEs in the study area posed significant noncarcinogenic risks for children. In terms of carcinogenic risk, the total TCR values of PTEs for adults and children were on the order of 1E−05, within the range of 1E−06 and 1E−04, indicating that the carcinogenic risk of soil PTEs for the human body is acceptable in the study area. In general, the noncarcinogenic and carcinogenic risks of PTEs from different sources in children were higher than those in adults, which can be explained by children having more opportunities PTE contamination through hand-to-mouth ingestion and dermal contact than adults due to the areas where they play and unhealthy eating habits (e.g., children are more likely to suck their fingers)^79^. Therefore, different parameters were set when employing the health risk assessment model.

**Table 4:** Specific noncarcinogenic risks of PTEs from different sources.

			As	Cd	Co	Cu	Ni	Pb	Sb	Sn	Tl	V	Zn	THI
Aksu Oasis	F1	Children	2.88E−02	6.96E−04	1.05E−01	2.20E−03	4.68E−03	2.17E−02	1.71E−02	2.35E−07	4.55E−02	1.92E−02	7.68E−04	2.46E−01
		Adults	7.14E−03	1.68E−04	2.56E−02	5.27E−04	1.12E−03	6.54E−03	5.01E−03	5.95E−08	1.06E−02	4.48E−03	1.87E−04	6.13E−02
	F2	Children	1.30E−01	5.67E−04	1.22E−01	1.39E−03	4.20E−03	5.18E−02	1.15E−02	2.13E−05	2.09E−01	2.21E−02	6.88E−04	5.53E−01
		Adults	3.22E−02	1.37E−04	2.98E−02	3.32E−04	1.01E−03	1.56E−02	3.37E−03	5.41E−06	4.87E−02	5.14E−03	1.67E−04	1.36E−01
	F3	Children	1.88E−01	8.65E−05	2.17E−02	2.41E−04	0.00E+00	1.70E−02	2.34E−02	1.19E−05	7.48E−02	5.63E−03	3.50E−04	3.31E−01
		Adults	4.67E−02	2.08E−05	5.27E−03	5.77E−05	0.00E+00	5.12E−03	6.87E−03	3.02E−06	1.74E−02	1.31E−03	8.52E−05	8.29E−02
	Total	Children	3.46E−01	1.35E−03	2.49E−01	3.83E−03	8.89E−03	9.06E−02	5.20E−02	3.35E−05	3.29E−01	4.69E−02	1.81E−03	1.13E+00
		Adults	8.60E−02	3.25E−04	6.06E−02	9.16E−04	2.13E−03	2.73E−02	1.53E−02	8.49E−06	7.68E−02	1.09E−02	4.39E−04	2.81E−01
Hotan Oasis	Total	Children	3.09E−01	1.05E−03	2.31E−01	3.34E−03	8.90E−03	8.97E−02	3.56E−02	3.99E−05	3.94E−01	4.60E−02	1.62E−03	1.12E+00
		Adults	7.67E−02	2.54E−04	5.63E−02	8.00E−04	2.14E−03	2.70E−02	1.04E−02	1.01E−05	9.18E−02	1.07E−02	3.95E−04	2.76E−01
Yarkant River Oasis	F1	Children	1.05E−01	6.75E−04	1.45E−01	2.22E−03	5.77E−03	4.77E−02	2.19E−02	1.56E−05	1.93E−01	2.86E−02	1.04E−03	5.51E−01
		Adults	2.59E−02	1.63E−04	3.52E−02	5.30E−04	1.39E−03	1.44E−02	6.43E−03	3.95E−06	4.51E−02	6.66E−03	2.54E−04	1.36E−01
	F2	Children	2.46E−01	5.28E−04	8.36E−02	9.82E−04	2.30E−03	5.19E−02	1.71E−02	2.07E−05	1.72E−01	1.78E−02	6.10E−04	5.93E−01
		Adults	6.11E−02	1.27E−04	2.03E−02	2.35E−04	5.52E−04	1.56E−02	5.01E−03	5.24E−06	4.01E−02	4.14E−03	1.48E−04	1.47E−01
	Total	Children	3.51E−01	1.20E−03	2.28E−01	3.20E−03	8.07E−03	9.96E−02	3.90E−02	3.62E−05	3.66E−01	4.64E−02	1.65E−03	1.14E+00
		Adults	8.71E−02	2.90E−04	5.56E−02	7.65E−04	1.94E−03	3.00E−02	1.14E−02	9.19E−06	8.52E−02	1.08E−02	4.02E−04	2.83E−01
Kashgar Oasis	F1	Children	1.13E−01	4.24E−04	1.23E−01	1.68E−03	4.17E−03	3.62E−02	1.76E−02	1.08E−05	1.30E−01	2.43E−02	7.35E−04	4.52E−01
		Adults	2.82E−02	1.02E−04	2.99E−02	4.03E−04	1.00E−03	1.09E−02	5.18E−03	2.75E−06	3.04E−02	5.65E−03	1.79E−04	1.12E−01
	F2	Children	1.27E−01	2.44E−04	8.33E−02	1.12E−03	2.42E−03	3.95E−02	1.67E−02	1.69E−05	1.82E−01	1.57E−02	5.57E−04	4.69E−01
		Adults	3.15E−02	5.88E−05	2.03E−02	2.68E−04	5.81E−04	1.19E−02	4.89E−03	4.29E−06	4.24E−02	3.66E−03	1.35E−04	1.16E−01
	F3	Children	9.73E−02	5.53E−04	6.98E−02	1.28E−03	3.08E−03	2.98E−02	1.07E−02	6.68E−06	7.46E−02	1.21E−02	6.26E−04	3.00E−01
		Adults	2.41E−02	1.33E−04	1.70E−02	3.06E−04	7.41E−04	8.95E−03	3.14E−03	1.69E−06	1.74E−02	2.83E−03	1.52E−04	7.48E−02
	Total	Children	3.38E−01	1.22E−03	2.76E−01	4.08E−03	9.67E−03	1.05E−01	4.50E−02	3.44E−05	3.87E−01	5.21E−02	1.92E−03	1.22E+00
		Adults	8.39E−02	2.94E−04	6.71E−02	9.77E−04	2.32E−03	3.17E−02	1.32E−02	8.73E−06	9.02E−02	1.21E−02	4.67E−04	3.02E−01

**Table 5:** Specific carcinogenic risks of PTEs from different sources.

Aksu Oasis		As	Ni	Pb	TCR	Kashgar Oasis		As	Ni	Pb	TCR
F1	Children	1.11E−06	2.08E−05	1.98E−09	2.19E−05	F1	Children	4.38E−06	1.85E−05	3.3E−09	2.29E−05
	Adults	1.1E−06	2.28E−05	1.82E−09	2.39E−05		Adults	4.35E−06	2.03E−05	3.03E−09	2.47E−05
F2	Children	5E−06	1.87E−05	4.72E−09	2.37E−05	F2	Children	4.9E−06	1.07E−05	3.59E−09	1.56E−05
	Adults	4.96E−06	2.05E−05	4.35E−09	2.55E−05		Adults	4.86E−06	1.18E−05	3.31E−09	1.66E−05
F3	Children	7.26E−06	0	1.55E−09	7.26E−06	F3	Children	3.75E−06	1.37E−05	2.71E−09	1.74E−05
	Adults	7.21E−06	0	1.43E−09	7.21E−06		Adults	3.73E−06	1.5E−05	2.49E−09	1.88E−05
Total	Children	1.34E−05	3.94E−05	8.25E−09	5.28E−05	Total	Children	1.3E−05	4.29E−05	9.6E−09	5.59E−05
	Adults	1.33E−05	4.33E−05	7.6E−09	5.66E−05		Adults	1.29E−05	4.71E−05	8.84E−09	6.01E−05

Yarkant River Oasis		As	Ni	Pb	TCR	Hotan Oasis		As	Ni	Pb	TCR
F1	Children	4.03E−06	2.56E−05	4.35E−09	2.96E−05	Total	Children	1.19E−05	3.95E−05	8.17E−09	5.14E−05
	Adults	4E−06	2.81E−05	4E−09	3.21E−05		Adults	1.18E−05	4.34E−05	7.52E−09	5.52E−05
F2	Children	9.5E−06	1.02E−05	4.73E−09	1.97E−05						
	Adults	9.43E−06	1.12E−05	4.35E−09	2.06E−05						
Total	Children	1.35E−05	3.58E−05	9.07E−09	4.93E−05						
	Adults	1.34E−05	3.93E−05	8.35E−09	5.27E−05

In terms of the proportion of human health risks caused by PTEs from different sources (Fig. 6), those from mixed sources, which were considered to have the largest contribution to PTEs in the Aksu Oasis, Kashgar Oasis and Yarkant River Oasis, were not the largest. This might be explained by the presence of more toxic elements, such as As and Tl, in other sources of the three oases contributing the most (Fig. 3), while their low *R*_*f*_*D* may explain their greater noncarcinogenic risk. PTEs from agricultural activities and natural sources accounted for the largest proportion of carcinogenic risk in the Aksu Oasis, while mixed sources accounted for the largest proportion of carcinogenic risk in the Kashgar Oasis and Yarkant River Oasis. This was mainly because the presence of As, Cd, Co, Ni and Pb elements with carcinogenic risk in the above two sources accounted for a large proportion (Fig. 3), thus posing a large carcinogenic risk. The results of the proportion of health risks attributed to different sources of PTEs to the human body showed that the control of human health risks of PTEs cannot be determined based on their concentration alone. Therefore, in view of the obvious noncarcinogenic risk of soil PTEs in the four oases to children, from the results of the contribution of different sources of PTEs to the noncarcinogenic risk to children, agricultural activities and natural sources, industrial sources and atmospheric dust fall were the priority control sources in the Aksu Oasis, Kashgar Oasis and Yarkant River Oasis, respectively.

## Discussion

As the analysis results of this study show, the pollution characteristic common to the four oases in the source region of the Tarim River was that Cd was the PTE with the most serious pollution level. Cd is the most common pollutant in agricultural soil in China^80,81^. Studies have shown that the concentration of Cd in agricultural soil in China is significantly correlated with the amount of fertilizer (N, P, K, and compound fertilizer) (*p* < 0.05)^80,82^. In this study, the source analysis results for soil PTEs of the four oases in the source region of the Tarim River also showed that Cd was closely related to agricultural activities (Supplementary Table S5). Therefore, the input of agricultural activities was the source of PTEs that need to be controlled in oases in this region. The difference in PTE pollution characteristics among the four oases was that those of the Yarkant River Oasis, Kashgar Oasis and Aksu Oasis were similar, while the PTEs in the agricultural soil of the Hotan Oasis were most affected by human activities, which indicates that the Hotan Oasis can be used as the key area for soil PTE control in the source region of the Tarim River.

Therefore, specific measures are proposed for the safe management of agricultural soil in the oases in the source region of the Tarim River: (1) It is essential to effectively control and optimize agricultural activities. The planting of crops requires fertile soil, but unfortunately, the excessive application of chemical fertilizers and pesticides has placed a great burden on the soil, and a large number of PTEs have accumulated^83^. Therefore, it is necessary to reduce the environmental pressure on soil by developing organic and ecological agriculture and reducing the use of chemical fertilizers and pesticides. (2) Because the oasis is adjacent to the desert, a large amount of sand/dust is incorporated into the soil through atmospheric dust fall, which is characteristic of the agricultural soil material composition in oases in arid areas. Therefore, it is necessary to strengthen soil and atmospheric dust monitoring in the Hotan Oasis, the key area of ecological risk assessment, to minimize the ecological risk caused by soil PTEs and the noncarcinogenic health risk incurred through hand-to-mouth ingestion and dermal contact. In addition, the ecological risks of specific sources and the key sources of PTEs will change with the seasons^16^, so long-term continuous monitoring is also necessary. (3) In view of the high ecological risk caused by PTEs in soil, it is necessary to pay attention to the migration and transformation of potential toxic elements in the soil-crop system and to control their accumulation in the food chain. Adjusting the agricultural structure of key regions can promote the sustainable development of agriculture and the economy from the perspective of geochemistry.

This study still has some limitations and needs further study. First, although the Geodetector model was included in the source analysis of PTEs in soil, due to the availability of data, the number of environmental variables included in this study was limited could not fully reflect the specific source of PTEs. Moreover, the mechanism underlying interactions between environmental variables (such as pH, *ST* and *TN*) and PTEs in soil is unknown^84^. Therefore, future research should further explore the potential source analysis of soil PTEs. Second, the parameters used in the human health risk assessment model in this study were taken directly from relevant documents, which increased the uncertainty of the assessment results to a certain extent. Moreover, there may be synergistic or antagonistic effects between various potentially toxic elements^85,86^, resulting in joint toxicity and increasing the error of health risk assessment results for individual elements. Finally, in the field survey of this study, it was found that the sand and dust in the oases in the source area of the Tarim River (especially Hotan Oasis) was relatively serious. Since the PTEs in dust fall can enter plants through the leaves and then enter the human body through the food chain^87^, the health risk results of this study may be limited by missing information regarding the intake route. Further research is recommended.

## Conclusions

In this study, the GBVs and pollution levels of PTEs in oasis agricultural soils of the source region of the Tarim River were revealed, source apportionment was explored, and the key sources and risk areas were identified considering the source proportions and source risks. The following conclusions were reached: (1) According to the GBVs and pollution index results, Cd was the most strongly polluting PTE in agricultural soils in the four oases. The PLI and PN index showed that the pollution level of PTEs in agricultural soil in the Hotan Oasis was significantly higher than that in the other three oases. (2) The source analysis results obtained by combining multivariate statistical analysis, the PMF model and the Geodetector model showed that the sources of PTEs in agricultural soil of the four oases in the source area of the Tarim River were different. (3) The ecological risk caused by PTEs of agricultural soil in the Hotan Oasis was at a medium level, and the PTEs in the other three oases posed no ecological risk. In terms of health risk, soil PTEs in the four regions posed an obvious noncarcinogenic risk for children, and the TCR for adults and children was within the range of 1E-06 and 1E-04, indicating that the carcinogenic risk was acceptable. (4) It is suggested that Hotan Oasis be treated as a key prevention and control area and its agricultural activities and atmospheric dust fall be controlled. The priority control sources of the Aksu Oasis, Kashgar Oasis and Yarkant River Oasis were agricultural activities and natural sources, industrial sources and atmospheric dust fall, respectively. The results of this study can provide policy makers with scientific information on environmental risk management strategies for agricultural soils to reduce ecological and health risks from PTEs.

## Supplementary Information

Supplementary Information.

## Data Availability

The datasets generated and/or analyzed during the current study are not publicly available because the data are a part of an ongoing study, but they are available from the corresponding author on reasonable request.

## References

[1] FAO *et al*. Status of the world’s soil resources (SWSR): Main Report. In *2015, Food and Agriculture Organization of the United Nations and Intergovernmental Technical Panel on Soils*. Rome.

[2] Zhao K (2020). Risk assessment, spatial patterns and source apportionment of soil heavy metals in a typical Chinese hickory plantation region of southeastern China. Geoderma.

[3] Liu W (2021). Potentially toxic elements in oasis agricultural soils caused by high-intensity exploitation in the piedmont zone of the Tianshan mountains, China. Agric. Basel.

[4] Guo X (2021). Multi-level methods to quantify risk assessment, source apportionment and identifying key risk areas of soil toxic elements in Ashi River watershed, China. Sci. Total Environ..

[5] Esmaeilzadeh M (2021). Contamination and ecological risk assessment of trace elements in sediments of the Anzali Wetland, Northern Iran. Water Sci. Technol..

[6] Wu M (2021). Heavy metal pollution from copper smelting during the Shang Dynasty at the Laoniupo site in the Bahe River valley, Guanzhong Basin, China. J. Geograph. Sci..

[7] Taspinar K (2021). Soil contamination and healthy risk assessment of peach orchards soil of Bilecik Province Turkey. Int. J. Environ. Health Res..

[8] Tian K (2017). Geochemical baseline establishment and ecological risk evaluation of heavy metals in greenhouse soils from Dongtai, China. Ecol. Indic..

[9] Karim Z (2015). Geochemical baseline determination and pollution assessment of heavy metals in urban soils of Karachi, Pakistan. Ecol. Indic..

[10] Wang S (2019). Geochemical baseline establishment and pollution source determination of heavy metals in lake sediments: A case study in Lihu Lake, China. Sci. Total Environ..

[11] Galan E (2008). Influence of geological setting on geochemical baselines of trace elements in soils. Application to soils of South-West Spain. J. Geochem. Explor..

[12] Meklit T (2009). Combining marginal and spatial outliers identification to optimize the mapping of the regional geochemical baseline concentration of soil heavy metals. Geoderma.

[13] Li Z (2019). Geochemical baseline values determination and evaluation of heavy metal contamination in soils of lanping mining valley (Yunnan Province, China). Int. J. Environ. Res. Public Health.

[14] Mao L (2022). Improved geochemical baseline establishment based on diffuse sources contribution of potential toxic elements in agricultural alluvial soils. Geoderma.

[15] Liu J (2018). Quantitative contributions of the major sources of heavy metals in soils to ecosystem and human health risks: A case study of Yulin, China. Ecotoxicol. Environ. Saf..

[16] Men C (2020). Source-specific ecological risk analysis and critical source identification of heavy metals in road dust in Beijing, China. J. Hazard. Mater..

[17] Taghvaee S (2018). Source-specific lung cancer risk assessment of ambient PM2.5-bound polycyclic aromatic hydrocarbons (PAHs) in central Tehran. Environ. Int..

[18] Chen R (2021). Source-specific health risk assessment of PM2.5-bound heavy metals based on high time-resolved measurement in a Chinese megacity: Insights into seasonal and diurnal variations. Ecotoxicol. Environ. Saf..

[19] Guo G (2021). Source-specific ecological and health risks of potentially toxic elements in agricultural soils in Southern Yunnan Province and associated uncertainty analysis. J. Hazard. Mater..

[20] Xia F (2021). Integrated source-risk and uncertainty assessment for metals contamination in sediments of an urban river system in eastern China. CATENA.

[21] Yang Y (2017). Space-time quantitative source apportionment of soil heavy metal concentration increments. Environ. Pollut..

[22] Jin G (2019). Source apportionment of heavy metals in farmland soil with application of APCS-MLR model: A pilot study for restoration of farmland in Shaoxing City Zhejiang, China. Ecotoxicol. Environ. Saf..

[23] Ye C (2011). Assessing soil heavy metal pollution in the water-level-fluctuation zone of the Three Gorges Reservoir, China. J. Hazard. Mater..

[24] Bhuiyan MAH (2021). Enrichment, sources and ecological risk mapping of heavy metals in agricultural soils of dhaka district employing SOM, PMF and GIS methods. Chemosphere.

[25] Shi T (2022). Machine learning can identify the sources of heavy metals in agricultural soil: A case study in northern Guangdong Province, China. Ecotoxicol. Environ. Saf..

[26] Wang S (2022). Concentrations, spatial distribution, sources and environmental health risks of potentially toxic elements in urban road dust across China. Sci. Total Environ..

[27] Yang Y (2020). Beyond mere pollution source identification: Determination of land covers emitting soil heavy metals by combining PCA/APCS, GeoDetector and GIS analysis. CATENA.

[28] Zhao Y (2020). Cadmium source identification in soils and high-risk regions predicted by geographical detector method. Environ. Pollut..

[29] Zhao X (2015). Water use efficiency in saline soils under cotton cultivation in the Tarim River Basin. Water.

[30] Wang F (2019). Assessment of the irrigation water requirement and water supply risk in the Tarim River Basin, Northwest China. Sustainability.

[31] Li W (2022). Analysis of the consequences of land-use changes and soil types on organic carbon storage in the Tarim River Basin from 2000 to 2020. Agric. Ecosyst. Environ..

[32] Xue L (2019). Spatiotemporal analysis of ecological vulnerability and management in the Tarim River Basin, China. Sci. Total Environ..

[33] Sun Y (2018). Assessment of heavy metal (metalloid) pollution and potential ecological risk for farmland soil in Yutian County of Xinjiang. Xinjiang Agric. Sci..

[34] Gu S (2019). Characteristics and ecological risk assessment of heavy metal pollution in farmland soil in Minfeng county of Xinjiang. J. Arid Land Resour. Environ..

[35] Zeng Y (2017). Distribution characteristics and assessment for farmland soil heavy metals pollution in Ruoqiang County of Xinjiang. J. Arid Land Resour. Environ..

[36] Mamut A (2018). Pollution and ecological risk assessment of heavy metals in farmland soils in Yanqi County, Xinjiang, Northwest China. Eurasian Soil Sci..

[37] Mamattursun E (2017). Assessment of heavy metal pollution and its potential ecological risks of farmland soils of oasis in Bosten Lake Basin. Acta Geograph. Sin..

[38] Mamut A (2017). The spatial distribution, contamination, and ecological risk assessment of heavy metals of farmland soils in Karashahar-Baghrash oasis, northwest China. Hum. Ecol. Risk Assess..

[39] Fan W (2021). Heavy metal pollution and health risk assessment of agricultural land in the Southern Margin of Tarim Basin in Xinjiang, China. Int. J. Environ. Health Res..

[40] Li Y (2009). Rehabilitating China's Largest Inland River. Conserv. Biol..

[41] Zhang B (2019). Comparative analysis of the spatial pattern of settlements between the source oasis and the terminal oasis of the Tarim River. Jiangsu Agric. Sci..

[42] Jiang LW (2005). Water resources, land exploration and population dynamics in arid areas—The case of the Tarim River Basin in Xinjiang of China. Popul. Environ..

[43] Huang S (2018). Adaptation strategies of agriculture and water management to climate change in the Upper Tarim River basin, NW China. Agric. Water Manag..

[44] Ma H (2012). The analysis of environmental impacts of chemical fertilizer in Tarim River Basin. Chin. Agric. Sci. Bull..

[45] Wei C (2012). Geochemical baselines of heavy metals in the sediments of two large freshwater lakes in China: Implications for contamination character and history. Environ. Geochem. Health.

[46] Jiang H (2020). An integrated approach to quantifying ecological and human health risks from different sources of soil heavy metals. Sci. Total Environ..

[47] Yan Y (2022). A comprehensive analysis on source-specific ecological risk of metal(loid)s in surface sediments of mangrove wetlands in Jiulong River Estuary, China. CATENA.

[48] Hu Y (2020). Quantitative source apportionment of heavy metal(loid)s in the agricultural soils of an industrializing region and associated model uncertainty. J. Hazard. Mater..

[49] Sun L (2019). Levels, sources, and spatial distribution of heavy metals in soils from a typical coal industrial city of Tangshan, China. CATENA.

[50] Lv J (2019). Multivariate receptor models and robust geostatistics to estimate source apportionment of heavy metals in soils. Environ. Pollut..

[51] Sheng D (2022). Contamination characteristics, source identification, and source-specific health risks of heavy metal(loid)s in groundwater of an arid oasis region in Northwest China. Sci. Total Environ..

[52] Liu H (2014). Heavy metal pollution feature analysis and potential ecological risk assessment of the coalbed methane production on the topsoil quality of the mining area. J. Saf. Environ..

[53] Liu Y (2018). Calculation of Thallium's toxicity coefficient in the evaluation of potential ecological risk index: A case study. Chemosphere.

[54] USEPA.* Exposure Factors Handbook*, 2011 ed. (Final). (U.S. Environmental Protection Agency, 2011).

[55] USEPA. *Exposure Factors Handbook, Volume 1: General Factors*. (U.S, Environmental Protection Agency, Office of Research and Development, 1997).

[56] Zhang H (2021). Pollutant source, ecological and human health risks assessment of heavy metals in soils from coal mining areas in Xinjiang, China. Environ. Res..

[57] Shi X (2022). Contamination and source-specific risk analysis of soil heavy metals in a typical coal industrial city, central China. Sci. Total Environ..

[58] Ferreira-Baptista L (2005). Geochemistry and risk assessment of street dust in Luanda, Angola: A tropical urban environment. Atmos. Environ..

[59] Dong B (2019). Multiple methods for the identification of heavy metal sources in cropland soils from a resource-based region. Sci. Total Environ..

[60] Guo B (2020). Health risk assessment of heavy metal pollution in a soil-rice system: A case study in the Jin-Qu Basin of China. Sci. Rep..

[61] Lei L (2015). Human health risk assessment of heavy metals in the irrigated area of Jinghui, Shaanxi, China, in terms of wheat flour consumption. Environ. Monit. Assess..

[62] Guan Q (2018). Contamination levels and health risk assessments of heavy metals in an oasis-desert zone: A case study in northwest China. Environ. Sci. Pollut. Res..

[63] Guan Q (2022). Probabilistic risk assessment of heavy metals in urban farmland soils of a typical oasis city in northwest China. Sci. Total Environ..

[64] Baghdady A (2018). Assessment of metal contamination and natural radiation hazards in different soil types near iron ore mines, Bahariya Oasis, Egypt. Arab. J. Geosci..

[65] CNEMC. *Soil Elements Background Values in China*. (China National Environmental Monitoring Centre, 1990).

[66] Eziz M (2018). Soil heavy metal pollution and ecological risk warning assessment of pepper field in Yanqi Basin, Xinjiang. Acta Ecol. Sin..

[67] Fusheng W (1991). Study on the background value of soil environment in China. Environ. Sci..

[68] Sun D (2021). Determination of heavy metals geochemical baseline value in paddy fields in the Dumu River Basin of Longli Coal Mine in Guizhou. Earth Environ..

[69] Yao R (2021). Response of soil characteristics and bacterial communities to nitrogen fertilization gradients in a coastal salt-affected agroecosystem. Land Degrad. Dev..

[70] Wen Q (2002). Study on dustfall and its effect on soils in Hotan, Xinjiang. Arid Zone Res..

[71] Zhong Q (2022). Thallium isotopic compositions as tracers in environmental studies: A review. Environ. Int..

[72] Cai L-M (2019). Heavy metals in agricultural soils from a typical township in Guangdong Province, China: Occurrences and spatial distribution. Ecotoxicol. Environ. Saf..

[73] Cheng W (2020). Geographic distribution of heavy metals and identification of their sources in soils near large, open-pit coal mines using positive matrix factorization. J. Hazard. Mater..

[74] Thorpe A (2008). Sources and properties of non-exhaust particulate matter from road traffic: A review. Sci. Total Environ..

[75] Martinez J (2008). Soil contamination from urban and industrial activity: Example of the mining district of Linares (southern Spain). Environ. Geol..

[76] Li X (2020). Spatial variation and source identification of heavy metals in sediments in Shaanxi section of Weihe River, Northwest China. Yingyong Shengtai Xuebao.

[77] Jiang H (2021). An integrated exploration on health risk assessment quantification of potentially hazardous elements in soils from the perspective of sources. Ecotoxicol. Environ. Saf..

[78] Yu H (2019). A seriously sand storm mixed air-polluted area in the margin of Tarim Basin: Temporal-spatial distribution and potential sources. Sci. Total Environ..

[79] Huang C (2022). Quantitative analysis of ecological risk and human health risk of potentially toxic elements in farmland soil using the PMF model. Land Degrad. Dev..

[80] Shi T (2019). Status of cadmium accumulation in agricultural soils across China (1975–2016): From temporal and spatial variations to risk assessment. Chemosphere.

[81] Zhao F-J (2015). Soil contamination in China: Current status and mitigation strategies. Environ. Sci. Technol..

[82] NSPCIR *et al.**The National Soil Pollution Condition Investigation Report*. (2014).

[83] Qin G (2021). Soil heavy metal pollution and food safety in China: Effects, sources and removing technology. Chemosphere.

[84] Zou B (2017). An integrated H-G scheme identifying areas for soil remediation and primary heavy metal contributors: A risk perspective. Sci. Rep..

[85] Zeng Q-B (2014). The combined effects of fluorine and arsenic on renal function in a Chinese population. Toxicol. Res..

[86] Jadhav SV (2015). Arsenic and fluoride contaminated groundwaters: A review of current technologies for contaminants removal. J. Environ. Manag..

[87] Gao J (2021). Assessment of the pollution levels of potential toxic elements in urban vegetable gardens in southwest China. Sci. Rep..

