# Supplementary material for: Source apportionment and source-specific risk evaluation of potential toxic elements in oasis agricultural soils of Tarim River Basin
Source: Sci Rep. 2023 Feb 20;13:2980. doi: 10.1038/s41598-023-29911-3 (PMC9941508; doi:10.1038/s41598-023-29911-3)
Supplement: Supplementary file 1 — Supplementary Information. [file 41598_2023_29911_MOESM1_ESM.docx]

**Source apportionment and source-specific risk evaluation of potential toxic elements in oasis agricultural soils of Tarim River Basin**

**Yizhen Li^1,2,3^, Jilili Abuduwaili^1,2,3^*, Long Ma^1,2,3^, Wen Liu^1,2,3^, Tao Zeng^1,2,3^**

^1^ State Key Laboratory of Desert and Oasis Ecology, Xinjiang Institute of Ecology and Geography, Chinese Academy of Sciences, Urumqi 830011, China

^2^ Research Center for Ecology and Environment of Central Asia, Chinese Academy of Sciences, Urumqi 830011, China

^3^ University of Chinese Academy of Sciences, Beijing 100049, China

*** Correspondence to** [**jilil@ms.xjb.ac.cn**](mailto:jilil@ms.xjb.ac.cn)**.**

**Supplementary Material S1**

**PMF Model:**

The initial matrix data (X_n×m_) was decomposed into two matrices, including source contribution matrix (G_n×p_) and the source profile matrix F (G_p×m_) (USEPA, 2011a). It can be expressed as follows:

$x_{ij}=\sum_{k=1}^{p} g_{ik}f_{kj}+e_{ij}$ (1)

Where, *i* is the series of the samples, *j* is the PTEs determined, *p* is the number of potential sources, *x_i_*_j_ is the concentration of the *j*th PTE in the *i*th sample (mg/kg); *g_ik_* is the contribution of the *k*th source in the *i*th sample; *f_kj_* is the amount of the *j*th PTE from the *k*th source factor; *p* represents the number of source factors; and *e_ij_* is the residual.

The concentration and uncertainty of each PTE are input into the PMF Model. The uncertainty (u) of the PTEs was calculated as follows:

$u_{ij}=\frac{5}{6}\times MDL, x_{ij}\leq MDL$ (2)

$u_{ij}=\sqrt{({\sigma\times x_{ij})}^{2}+{(MDL)}^{2}} , x_{ij}>MDL$(3)

Where x_ij_ is the concentration of PTE, MDL is the method detection limit, and σ is the relative standard deviation of the concentration of PTEs.

Factor contributions and profiles can be determined by minimizing the objective function as follows: (USEPA, 2014)

$Q=\sum_{i=1}^{n} \sum_{j=1}^{m} {[\frac{x_{ij}-\sum_{k=1}^{p} g_{ik}f_{kj}}{u_{ij}}]}^{2}$ (4)

Where uij is the uncertainty of the *j*th PTE in the *i*th sample. Qtrue and Qrobust are two set data of Q used in the PMF model. The best simulated run was identified by the lowest Qrobust. The determination of the number of the factors is critical to the results of the PMF model.

**GeoDetector**

Geodetector is a tool that measures the degree of spatial differentiation of spatial variables and the explanatory power of their influencing factors(Wang and Xu, 2017). The factor detector quantifies the degree of interpretation of the spatial distribution of soil salinity by various natural and human factors(Wang and Xu, 2017):

 (5)

where h is the stratification of soil salinity impact factors, i.e., classification or zoning. N and N_h_ represent the number of samples for the entire study area and stratum h, respectively; whereas σ^2^ and σ^2^_h_ indicate the variance of soil samples in the study area and stratum h, respectively. SSW is the within sum of squares, and SST is the total sum of squares. The q values indicate the spatial heterogeneity of influencing factors on soil salinity, and its value ranges from 0 to 1.

Table S1. The longitude and latitude information of sampling points

| No. | N° | E° | No. | N° | E° | No. | N° | E° | No. | N° | E° |
| --- | --- | --- | --- | --- | --- | --- | --- | --- | --- | --- | --- |
| KS01 | 39.2792 | 75.5762 | YS01 | 38.3251 | 77.0201 | AS01 | 41.2395 | 79.2810 | HS01 | 37.3902 | 79.4976 |
| KS02 | 39.1037 | 75.6361 | YS02 | 38.1067 | 77.1866 | AS02 | 41.2701 | 79.4446 | HS02 | 37.4619 | 79.5967 |
| KS03 | 39.4569 | 75.6657 | YS03 | 38.4982 | 77.2459 | AS03 | 41.1871 | 80.2091 | HS03 | 37.3968 | 79.5988 |
| KS04 | 39.4910 | 75.8497 | YS04 | 38.2615 | 77.3555 | AS04 | 41.2473 | 80.1429 | HS04 | 37.2541 | 79.5743 |
| KS05 | 39.3571 | 75.8242 | YS05 | 37.8879 | 77.3075 | AS05 | 40.6744 | 81.7027 | HS05 | 37.1436 | 79.6671 |
| KS06 | 39.2545 | 75.8006 | YS06 | 38.7201 | 77.3609 | AS06 | 40.6502 | 80.3143 | HS06 | 37.2785 | 79.6712 |
| KS07 | 39.0886 | 75.7363 | YS07 | 38.5316 | 77.4802 | AS07 | 41.0374 | 80.2669 | HS07 | 37.3674 | 79.7507 |
| KS08 | 39.4246 | 75.9638 | YS08 | 38.9189 | 77.5357 | AS08 | 40.8133 | 80.3417 | HS08 | 37.0764 | 79.7278 |
| KS09 | 39.3136 | 75.9907 | YS09 | 38.7159 | 77.6087 | AS09 | 40.6359 | 79.8867 | HS09 | 37.1828 | 79.8313 |
| KS10 | 39.1106 | 76.2101 | YS10 | 39.1992 | 77.6953 | AS10 | 40.9008 | 80.2824 | HS10 | 37.2852 | 79.8154 |
| KS11 | 39.5446 | 76.0933 | YS11 | 38.9638 | 77.7325 | AS11 | 40.4715 | 80.3755 | HS11 | 37.2677 | 79.9086 |
| KS12 | 39.4079 | 76.1215 | YS12 | 39.3678 | 77.9448 | AS12 | 40.6254 | 80.8096 | HS12 | 37.0592 | 79.8849 |
| KS13 | 39.2412 | 76.1672 | YS13 | 39.3677 | 77.9446 | AS13 | 40.4313 | 80.8129 | HS13 | 36.9980 | 79.9600 |
| KS14 | 38.9783 | 76.2986 | YS14 | 39.0649 | 77.8811 | AS14 | 40.4983 | 81.1406 | HS14 | 37.1626 | 79.9887 |
| KS15 | 39.7073 | 77.2809 | YS15 | 39.4720 | 78.2117 | AS15 | 40.5965 | 81.5010 | HS15 | 37.0599 | 80.1551 |
| KS16 | 39.3915 | 76.3638 | YS16 | 39.6141 | 78.3677 | AS16 | 40.7578 | 80.5095 | HS16 | 37.1492 | 80.1754 |
| KS17 | 39.2884 | 76.3806 | YS17 | 39.7996 | 78.4689 |  |  |  |  |  |  |
| KS18 | 39.0769 | 76.4453 | YS18 | 39.7540 | 78.6543 |  |  |  |  |  |  |
| KS19 | 39.4626 | 76.5920 | YS19 | 37.9891 | 77.4916 |  |  |  |  |  |  |
| KS20 | 39.3012 | 76.5864 |  |  |  |  |  |  |  |  |  |
| KS21 | 39.1231 | 76.5971 |  |  |  |  |  |  |  |  |  |
| KS22 | 39.6818 | 76.7629 |  |  |  |  |  |  |  |  |  |
| KS23 | 39.5172 | 76.7991 |  |  |  |  |  |  |  |  |  |
| KS24 | 39.2762 | 76.7612 |  |  |  |  |  |  |  |  |  |
| KS25 | 39.1610 | 76.8985 |  |  |  |  |  |  |  |  |  |
| KS26 | 39.6961 | 76.9977 |  |  |  |  |  |  |  |  |  |
| KS27 | 39.7674 | 77.3471 |  |  |  |  |  |  |  |  |  |

Table S2 Classifications of potential ecological risk corresponding to PF、PIL、P_N_ and EIRI(Guo et al., 2021; Men et al., 2020)

| PF | Classification standard | PLI | Classification standard | P_N_ | Classification standard | EIRI | Classification standard |
| --- | --- | --- | --- | --- | --- | --- | --- |
| PF≤1 | Uncontaminated | PLI≤1 | Uncontaminated | P_N_≤0.7 | Uncontaminated | EIRI≤40 | Low risk |
| 1<PF≤2 | Slightly contaminated | 1<PLI≤2 | Moderate contaminated | 0.7<PN≤1 | Alert level | 40<EIRI≤80 | Moderate risk |
| 2<PF≤3 | Mild contaminated | 2<PLI≤5 | Highly contaminated | 1<PN≤2 | Slightly contaminated | 80<EIRI≤160 | Considerable risk |
| 3<PF≤5 | Moderate contaminated | PLI>5 | Extremely contaminated | 2<PN≤3 | Moderate contaminated | 160<EIRI≤320 | High risk |
| PF>5 | High contaminated | |  | PN>3 | Highly contaminated | EIRI>320 | Very high risk |

Table S3 Parameter definitions and reference values of health risk assessment model (Rani et al., 2019; USEPA, 2002; 2011b; 2013)

| Parameters | Description (unit) | Group | Values | Reference |
| --- | --- | --- | --- | --- |
| IngR | Ingestion rate of soils (mg/day) | children | 200 | (USEPA, 2011a) |
|  |  | adults | 100 |  |
| ED | Exposure duration (year) | children | 6 | (USEPA, 2011a) |
|  |  | adults | 24 |  |
| EF | Exposure frequency(day/year) | children | 350 | (USEPA, 2011a) |
|  |  | adults | 350 |  |
| BW | Average body weight (kg) | children | 29 | (Zhang et al., 2021) |
|  |  | adults | 63 |  |
| ABS | Dermal absorption factor | children | 0.03 (As), 0.1 (Pb), 0.001 (Cd), 0.01 (others) | (Wang et al., 2018) |
|  |  | adults |  |  |
| AT | Average time(day) | children | Carcinogenic: 365×70 | (USEPA, 2011a) |
|  |  | adults | Non-carcinogenic: 365×ED |  |
| SA | Exposed skin area (cm^2^) | children | 2700 | (USEPA, 2011a) |
|  |  | adults | 5700 |  |
| AF | Adherence factor (mg/cm^2^) | children | 0.2 | (USEPA, 2011a) |
|  |  | adults | 0.07 |  |
| PEF | Particle emission factor (m^3^/kg) | children | 1.36E+09 | (USEPA, 2002) |
|  |  | adults | 1.36E+09 |  |

Table S4 Values of *R_f_D* and *SF* of heavy metals(Cao et al., 2016; Guo et al., 2021; Ma et al., 2020; Shen et al., 2019)

|  | As | Cd | Co | Cu | Ni | Pb | Sb | Sn | Tl | V | Zn |
| --- | --- | --- | --- | --- | --- | --- | --- | --- | --- | --- | --- |
| RfDing | 3.00E-04 | 1.00E-03 | 3.00E-04 | 4.00E-02 | 2.00E-02 | 3.50E-03 | 4.00E-04 | 6.00E-01 | 1.00E-05 | 9.00E-03 | 3.00E-01 |
| RfDder | 1.23E-04 | 2.50E-05 | 6.00E-05 | 1.20E-02 | 5.40E-03 | 5.25E-04 | 8.00E-06 | 6.00E-02 | 1.00E-05 | 9.00E-03 | 6.00E-02 |
| SFing | 1.50E+00 |  |  |  | 1.70E+00 | 8.50E-04 |  |  |  |  |  |
| SFder | 3.66E+00 |  |  |  | 4.25E+01 |  |  |  |  |  |  |

Table S5 Factor detector q value of soil PTEs and influencing factors in the four oases

| q | PTEs | DF | DR | pH | ST | TN | Fine silty | Silty | Coarse silty |
| --- | --- | --- | --- | --- | --- | --- | --- | --- | --- |
| Yarkant River Oasis | As | 0.63 | 0.34 | 0.28 | 0.57 | 0.47 | 0.64 | 0.70 | 0.56 |
|  | Cd | 0.31 | 0.47 | 0.71 | 0.34 | 0.67 | 0.47 | 0.79 | 0.29 |
|  | Co | 0.57 | 0.76 | 0.53 | 0.69 | 0.64 | 0.76 | 0.47 | 0.56 |
|  | Cu | 0.52 | 0.76 | 0.57 | 0.75 | 0.62 | 0.85 | 0.51 | 0.68 |
|  | Ni | 0.43 | 0.61 | 0.45 | 0.51 | 0.40 | 0.77 | 0.24 | 0.63 |
|  | Pb | 0.53 | 0.57 | 0.38 | 0.24 | 0.63 | 0.74 | 0.45 | 0.59 |
|  | Sb | 0.57 | 0.57 | 0.63 | 0.72 | 0.84 | 0.71 | 0.59 | 0.43 |
|  | Sn | 0.49 | 0.59 | 0.40 | 0.50 | 0.48 | 0.74 | 0.55 | 0.70 |
|  | Tl | 0.57 | 0.59 | 0.42 | 0.29 | 0.62 | 0.88 | 0.34 | 0.56 |
|  | V | 0.52 | 0.70 | 0.63 | 0.79 | 0.72 | 0.81 | 0.56 | 0.70 |
|  | Zn | 0.51 | 0.68 | 0.74 | 0.80 | 0.75 | 0.71 | 0.61 | 0.64 |
| Kashgar Oasis | As | 0.82 | 0.41 | 0.37 | 0.15 | 0.41 | 0.26 | 0.23 | 0.44 |
|  | Cd | 0.46 | 0.15 | 0.70 | 0.19 | 0.52 | 0.18 | 0.53 | 0.23 |
|  | Co | 0.40 | 0.46 | 0.34 | 0.21 | 0.18 | 0.37 | 0.07 | 0.38 |
|  | Cu | 0.46 | 0.40 | 0.47 | 0.16 | 0.19 | 0.32 | 0.11 | 0.40 |
|  | Ni | 0.30 | 0.38 | 0.38 | 0.18 | 0.25 | 0.35 | 0.39 | 0.46 |
|  | Pb | 0.65 | 0.56 | 0.14 | 0.17 | 0.30 | 0.33 | 0.29 | 0.49 |
|  | Sb | 0.65 | 0.65 | 0.18 | 0.11 | 0.21 | 0.32 | 0.25 | 0.34 |
|  | Sn | 0.76 | 0.44 | 0.46 | 0.26 | 0.33 | 0.33 | 0.29 | 0.34 |
|  | Tl | 0.79 | 0.53 | 0.33 | 0.22 | 0.29 | 0.32 | 0.30 | 0.32 |
|  | V | 0.35 | 0.53 | 0.34 | 0.16 | 0.15 | 0.32 | 0.14 | 0.40 |
|  | Zn | 0.52 | 0.28 | 0.31 | 0.17 | 0.28 | 0.46 | 0.11 | 0.23 |
| Aksu Oasis | As | 0.43 | 0.62 | 0.48 | 0.30 | 0.86 | 0.55 | 0.47 | 0.51 |
|  | Cd | 0.76 | 0.55 | 0.73 | 0.43 | 0.80 | 0.61 | 0.58 | 0.67 |
|  | Co | 0.73 | 0.35 | 0.60 | 0.38 | 0.56 | 0.84 | 0.61 | 0.91 |
|  | Cu | 0.66 | 0.39 | 0.59 | 0.35 | 0.72 | 0.91 | 0.78 | 0.94 |
|  | Ni | 0.59 | 0.43 | 0.60 | 0.41 | 0.61 | 0.83 | 0.65 | 0.85 |
|  | Pb | 0.69 | 0.47 | 0.68 | 0.48 | 0.43 | 0.77 | 0.49 | 0.76 |
|  | Sb | 0.80 | 0.60 | 0.38 | 0.38 | 0.85 | 0.80 | 0.82 | 0.88 |
|  | Sn | 0.63 | 0.62 | 0.49 | 0.72 | 0.91 | 0.66 | 0.49 | 0.53 |
|  | Tl | 0.43 | 0.55 | 0.65 | 0.77 | 0.67 | 0.64 | 0.41 | 0.58 |
|  | V | 0.68 | 0.36 | 0.55 | 0.24 | 0.63 | 0.91 | 0.65 | 0.88 |
|  | Zn | 0.62 | 0.49 | 0.61 | 0.33 | 0.77 | 0.75 | 0.85 | 0.91 |
| Hotan Oasis | As | 0.25 | 0.44 | 0.82 | 0.28 | 0.59 | 0.91 | 0.79 | 0.78 |
|  | Cd | 0.34 | 0.19 | 0.54 | 0.21 | 0.77 | 0.81 | 0.71 | 0.51 |
|  | Co | 0.62 | 0.70 | 0.42 | 0.45 | 0.79 | 0.84 | 0.85 | 0.90 |
|  | Cu | 0.56 | 0.58 | 0.51 | 0.42 | 0.76 | 0.87 | 0.87 | 0.86 |
|  | Ni | 0.48 | 0.60 | 0.48 | 0.30 | 0.72 | 0.94 | 0.74 | 0.81 |
|  | Pb | 0.48 | 0.59 | 0.41 | 0.64 | 0.69 | 0.74 | 0.88 | 0.92 |
|  | Sb | 0.41 | 0.39 | 0.66 | 0.47 | 0.70 | 0.88 | 0.88 | 0.85 |
|  | Sn | 0.33 | 0.43 | 0.77 | 0.34 | 0.57 | 0.99 | 0.71 | 0.71 |
|  | Tl | 0.40 | 0.56 | 0.60 | 0.35 | 0.66 | 0.94 | 0.76 | 0.78 |
|  | V | 0.63 | 0.70 | 0.31 | 0.47 | 0.80 | 0.81 | 0.79 | 0.88 |
|  | Zn | 0.48 | 0.45 | 0.62 | 0.30 | 0.74 | 0.94 | 0.83 | 0.83 |

Fig. S1 Cumulative frequency curve of soil PTEs in the Aksu Oasis

Fig. S2 Cumulative frequency curve of soil PTEs in the Kashgar Oasis

Fig. S3 Cumulative frequency curve of soil PTEs in the Yarkant River Oasis

Fig. S4 Cumulative frequency curve of soil PTEs in the Hotan Oasis

(a) (b)

(c) (d)

Fig. S5 Correlation analysis of soil PTEs in oasis at the source of the Tarim River (a for Yarkant River Oasis, b for Kashgar Oasis, c for Aksu Oasis and d for Hotan Oasis)

Cao S., Duan X., Zhao X., et al., 2016. Health risks of children's cumulative and aggregative exposure to metals and metalloids in a typical urban environment in China[J]. Chemosphere. 147 404-411.

Guo G., Wang Y., Zhang D., et al., 2021. Source-specific ecological and health risks of potentially toxic elements in agricultural soils in Southern Yunnan Province and associated uncertainty analysis[J]. Journal of Hazardous Materials. 417.

Ma L., Xiao T., Ning Z., et al., 2020. Pollution and health risk assessment of toxic metal(loid)s in soils under different land use in sulphide mineralized areas[J]. Science of the Total Environment. 724.

Men C., Liu R., Xu L., et al., 2020. Source-specific ecological risk analysis and critical source identification of heavy metals in road dust in Beijing, China[J]. Journal of Hazardous Materials. 388.

Rani N., Sastry B.S., Dey K., 2019. Assessment of metal contamination and the associated human health risk from dustfall deposition: a study in a mid-sized town in India[J]. Environmental Science and Pollution Research. (22): 23173-23191.

Shen Q., Zhang L., Kimirei I.A., et al., 2019. Vertical physicochemical parameter distributions and health risk assessment for trace metals in water columns in eastern Lake Tanganyika, Tanzania[J]. Journal of Oceanology and Limnology. 37 (1): 134-145.

USEPA (2002) Risk-based concentration table, Washington DC: Philadelphia PA.

USEPA (2011a) Exposure Factors Handbook, 2011 ed. (Final). U.S. Environmental Protection Agency, Washington.

USEPA (2011b) Exposure Factors Handbook, Final ed., (EPA/600/R-09/052F), Washington DC: U.S.

USEPA (2013) Regional Screening Levels (RSLs) - Generic Tables, San Francisco, CA.

Wang J., Xu C., 2017. Geodetector: Principle and prospective[J]. Acta Geographica Sinica. 72 (1): 116-134.

Wang X., He S., Chen S., et al., 2018. Spatiotemporal Characteristics and Health Risk Assessment of Heavy Metals in PM2.5 in Zhejiang Province[J]. International Journal of Environmental Research and Public Health. 15 (4).

Zhang H., Zhang F., Song J., et al., 2021. Pollutant source, ecological and human health risks assessment of heavy metals in soils from coal mining areas in Xinjiang, China[J]. Environmental Research. 202.
